# The harm of class imbalance corrections for risk prediction models: illustration and simulation using logistic regression

**DOI:** 10.1093/jamia/ocac093

**Published:** 2022-06-10

**Authors:** Ruben van den Goorbergh, Maarten van Smeden, Dirk Timmerman, Ben Van Calster

**Affiliations:** Julius Center for Health Sciences and Primary Care, UMC Utrecht, Utrecht University, Utrecht, The Netherlands; Julius Center for Health Sciences and Primary Care, UMC Utrecht, Utrecht University, Utrecht, The Netherlands; Department of Development and Regeneration, KU Leuven, Leuven, Belgium; Department of Obstetrics and Gynecology, University Hospitals Leuven, Leuven, Belgium; Department of Development and Regeneration, KU Leuven, Leuven, Belgium; Department of Biomedical Data Sciences, Leiden University Medical Center, Leiden, The Netherlands; EPI-Center, KU Leuven, Leuven, Belgium

**Keywords:** class imbalance, logistic regression, calibration, synthetic minority oversampling technique, undersampling

## Abstract

**Objective:**

Methods to correct class imbalance (imbalance between the frequency of outcome events and nonevents) are receiving increasing interest for developing prediction models. We examined the effect of imbalance correction on the performance of logistic regression models.

**Material and Methods:**

Prediction models were developed using standard and penalized (ridge) logistic regression under 4 methods to address class imbalance: no correction, random undersampling, random oversampling, and SMOTE. Model performance was evaluated in terms of discrimination, calibration, and classification. Using Monte Carlo simulations, we studied the impact of training set size, number of predictors, and the outcome event fraction. A case study on prediction modeling for ovarian cancer diagnosis is presented.

**Results:**

The use of random undersampling, random oversampling, or SMOTE yielded poorly calibrated models: the probability to belong to the minority class was strongly overestimated. These methods did not result in higher areas under the ROC curve when compared with models developed without correction for class imbalance. Although imbalance correction improved the balance between sensitivity and specificity, similar results were obtained by shifting the probability threshold instead.

**Discussion:**

Imbalance correction led to models with strong miscalibration without better ability to distinguish between patients with and without the outcome event. The inaccurate probability estimates reduce the clinical utility of the model, because decisions about treatment are ill-informed.

**Conclusion:**

Outcome imbalance is not a problem in itself, imbalance correction may even worsen model performance.

## INTRODUCTION

When developing clinical prediction models for a binary outcome, the percentage of individuals with the event of interest (ie, the event fraction) is often much lower than 50%. When the frequency of individuals with and without the event is unequal, the term “class imbalance” is often used.^1^ Class imbalance has been identified as a problem for the development of prediction models, in particularly when the interest is in the classification of individuals into a high risk versus low risk group (“classifier”).^1–3^ Commonly suggested solutions to address class imbalance include some form of resampling to create an artificially balanced dataset for model training. Common approaches are random undersampling (RUS), random oversampling (ROS), and SMOTE (Synthetic Minority Oversampling Technique).^2–5^

The classification accuracy of a model that classifies individuals into a high risk versus low risk group is defined as the percentage of individuals that are either true positive (individuals that have the event and are either correctly classified as high risk) or true negative (individuals that do not have the event and are correctly classified as low risk). To illustrate the possible impact of class imbalance, consider a simple model that classifies everyone as low risk. Such a classifier yields a classification accuracy of 50% if the event fraction is 50% (balanced), but a classification accuracy of 99% if the event fraction is 1% (highly imbalanced). That imbalanced datasets can easily lead to high classification accuracy is often labeled as problematic. For instance, He and Garcia write “we find that classifiers tend to provide a severely imbalanced degree of accuracy, with the majority class having close to 100% accuracy and the minority class having accuracies of 0%–10%, for instance.”^2^ Fernandez and colleagues write “the truth is that classifiers … tend to have great accuracy for the majority class while obtaining poor results (closer to 0%) for the minority class.”^3^

We argue that the class imbalance is not a pervasive problem for prediction model development. First, the problem is specific to the classification accuracy measure. The limitations of focusing on classification accuracy as a measure of predictive performance is well known.^6^^,^^7^ Second, if we consider models that produce estimated probabilities of the event of interest, an adjustment of the classification threshold probability can be used to ensure adequate classification performance (ie, probability threshold to classify individuals as high risk does not have to be 0.5).^8^ A probability threshold to select individuals for a given treatment implies certain misclassification costs and should be determined using clinical considerations.^8^ If we use a probability threshold of 0.1 to classify individuals as high risk and suggest a specific treatment, this means that we accept to treat up to 10 individuals in order to treat 1 individual with the event: we accept up to 9 false positives, or unnecessary treatments, per true positive.^9–11^ As Birch and colleagues write, models should be able to accommodate differing attitudes regarding misclassification costs.^12^ The problem then shifts from class imbalance to probability calibration: the model’s probability estimates should be reliable in order to make optimal decisions. This raises the question how class imbalance methods affect calibration.

## OBJECTIVE

In this study, we investigate the performance of standard and penalized logistic regression models developed in datasets with class imbalance. We hypothesize (1) that imbalance correction methods distort model calibration by leading to probability estimates that are too high, and (2) that shifting the probability threshold has similar impact on sensitivity and specificity as the use of imbalance correction methods.

## MATERIALS AND METHODS

### Imbalance correction methods and logistic regression models

When using RUS, the size of the majority class (ie, the group of individuals with observed events or nonevents, whichever is larger) is reduced by discarding a random set of cases until the majority class has the same size as the minority class. When using ROS, the size of the minority class is increased by resampling cases from the minority class, with replacement, until the minority class has the same size as the majority class. This results in an artificially balanced dataset containing duplicate cases for the minority class. SMOTE is a form of oversampling that creates new, synthetic cases that are interpolations of the original minority class cases.^4^^,^^5^ The procedure is as follows: for every minority class case, the k nearest minority class neighbors in the predictor space are determined, based on the Euclidean distance. Then, the differences between the feature vector of the minority case and those of its k nearest neighbors are taken. These differences are then multiplied by a random number between 0 and 1 and added to the feature vector of the minority case. By creating synthetic data in this manner, there is more variation in the minority cases and hence, the models trained on this dataset may be less prone to overfitting than when trained on ROS data. We used *k* = 5 when implementing SMOTE. SMOTE is designed to work with continuous variables. For use with ordinal or categorical variables, one may either use rounding or use an adaptation of SMOTE for mixed variable types.

**Figure 1.**
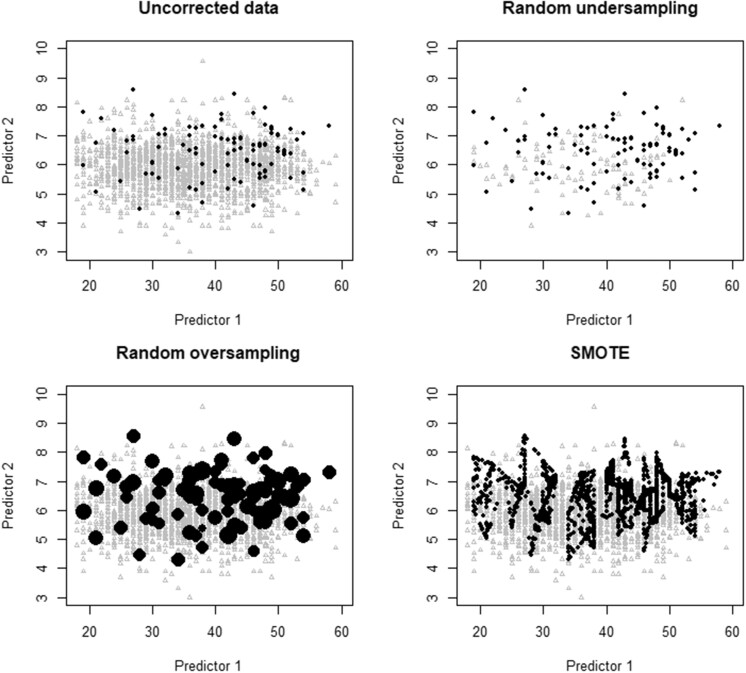
Visualization of how the imbalance correction methods work for a hypothetical dataset with 2 predictors. Black dots represent observations from the minority class, gray triangles represent observations from the majority class.

Figure 1 illustrates these methods using 2 predictors. The original dataset has 100 cases (red dots) with the event and 1900 without the event (gray triangles) (upper left panel). The difference between ROS (lower left panel) and SMOTE (lower right panel) is obvious. ROS includes many duplicates of the original cases from the minority class. SMOTE creates synthetic cases that lie on the “line” between 2 original minority cases. As lower sample size is well known to increase the risk of overfitting, we anticipated that RUS would require a larger sample size to perform well.^13–15^

Prediction models were developed using standard maximum likelihood logistic regression (SLR) and using penalized logistic regression with the ridge (or L2) penalty (Ridge).^16^ The lambda hyperparameter was tuned using a grid search based on 10-fold cross-validation.^17^ See Supplementary Material for details.

### Case study: estimating the probability of ovarian cancer

For illustration, we developed prediction models to estimate the probability of ovarian malignancy in premenopausal women presenting with at least 1 adnexal (ovarian, para-ovarian, or tubal) tumor. Prediction models for ovarian cancer diagnosis could be used to decide whether to operate and by whom (eg, whether referral to an experienced gynecological oncologist is warranted or not). We use data from consecutively recruited women across 3 waves (1999–2005, 2005–2007, and 2009–2012) of the International Ovarian Tumor Analysis (IOTA) study.^18^^,^^19^ We have ethics approval for secondary use of these data for methodological/statistical research (Research Ethics Committee University Hospitals KU Leuven, S64709). The study only included patients who were operated on, such that the reference standard (benign or malignant) could be based on histology. Borderline malignant tumors were considered malignant. Overall, 5914 patients were recruited across the 3 waves, of which 3369 premenopausal patients between 18 and 59 years. The prevalence of malignancy was 20% (658/3369), reflecting moderate imbalance.

We used the following predictors: age of the patient (years), maximum diameter of the lesion (mm), and number of papillary structures (ordinal variable with values 0 to 4; 4 referring to 4 or more papillary structures). To investigate performance of all models in combination with the different imbalance solutions, the data was first split randomly into a training set and a test set using a 4:1 ratio. This yielded a training dataset of size 2695 (518 events), and a test set of size 674 (140 events). The training set was either uncorrected or preprocessed using RUS, ROS, or SMOTE, resulting in 4 different datasets on which models were fitted: *D*_uncorrected_; *D*_RUS_; *D*_ROS_; and *D*_SMOTE_. Subsequently, prediction models were developed using SLR and Ridge, resulting in 4 (datasets) × 2 (algorithms) = 8 different models. To address potentially nonlinear associations with the outcome, continuous predictor variables were modeled using spline functions. In particular, we used restricted cubic splines with 3 knots.^20^ The resulting models were applied to the test set to obtain the model performance in terms of discrimination (the area under the ROC curve, AUROC), calibration (calibration intercept, calibration slope, flexible calibration curves), classification (accuracy, sensitivity, specificity), and clinical utility (Net Benefit) (Box).^10^^,^^11^^,^^21^^,^^22^ For classification, the “default” probability threshold of 0.5 was used as well as a probability threshold of 0.192 (518/2695, prevalence of malignancy in the training dataset) when class imbalance was not corrected.

Box. Test set performance domains and metrics.

1. **DISCRIMINATION** between cases with and without an event.The ***AUROC*** (area under the receiver operating characteristic curve) corresponds to the concordance statistic, and estimates the probability that a model gives a higher prediction for a random individual with the event than for a random individual without the event. The AUROC is 1 when a model gives higher predictions for all patients with an event than for all patients without an event, and 0.5 when the model cannot differentiate at all between patients with and without the event.
2. **CALIBRATION** or reliability of the predictions itself.The ***calibration intercept*** quantifies whether probability estimates are on average too high (overestimation, calibration intercept < 0) or too low (underestimation, calibration intercept > 0).^21^^,^^22^ It is calculated as the intercept a of the following logistic regression analysis on the test set: $\mathrm{logit}\left(P(Y=1)\right)=a+LP$, where LP is the logit of the estimated probability from the model. The LP is added as an offset, meaning that its coefficient is fixed at 1. When a is negative, the analysis indicates that we need to lower the predictions and hence that these predictions tended to be too high.The ***calibration slope*** quantifies whether probability estimates are too extreme (too close to 0 or 1, calibration slope < 1) or too modest (too close to the event fraction, calibration slope > 1).^21^^,^^22^ It is calculated as the coefficient b of the following logistic regression analysis on the test set: logitP(Y=1)=a’+b^*LP. When the slope (b) is <1, the analysis indicates that the predictions needed to be shrunk towards the average and hence were too extreme. Because we perform internal validation of performance, calibration slopes below 1 only reflect overfitting in this study.A flexible ***calibration curve*** visualizes the reliability of predictions conditional on the estimated probability.^22^ It is based on the following flexible logistic regression model on the test set: $\mathrm{logit}\left(P(Y=1)\right)=a’’+f(LP)$. The flexible function f(.) was based on loess (locally estimated scatter plot smoothing).
3. **CLASSIFICATION** of patients into high-risk and low-risk groups after specifying a risk threshold.The ***accuracy*** is the proportion of patients that are classified correctly, that is, the proportion of patients that are either true positives or true negatives: (TP + TN)/N, with TP the number of true positives, TN the number of true negatives, and N the sample size. The ***sensitivity*** (also known as recall) is the proportion of patients with the event that are classified as high risk: TP/(TP + FN), with FN the number of false negatives. The ***specificity*** is the proportion of patients without the event that are classified as low risk: TN/(TN + FP), with FP the number of false positives.
4. **CLINICAL UTILITY** of treatment decisions while taking misclassification costs into account.We assume that we use a model to identify high risk patients (estimated probability ≥ *t*), for which a given clinical intervention is warranted. ***Net Benefit*** quantifies the utility of the model to make such treatment decisions.^10^^,^^11^ It exploits the link between the risk threshold *t* and misclassification costs. For example, using *t *=* *0.1 means that we accept to treat at most 10 patients per true positive.^9^ In other words, we tolerate 9 false positives (unnecessary treatments) per true positive (necessary treatment). So as long as you have <9 false positives per true positive, the benefits outweigh the harms. Net Benefit is therefore calculated as $\left(TP-\frac{t}{1-t}FP\right)/N$. Net Benefit can be calculated for several potential values for *t*. A plot of Net Benefit for a range of thresholds is a decision curve. Net Benefit can also be calculated for 2 default strategies: treating everyone or treating no one. Treating no one has a Net Benefit of 0 by definition. Treating everyone has a positive Net Benefit when misclassification costs clearly favor true positives (*t* is low). If, for a given *t*, the Net Benefit of a model is not higher than the Net Benefit of the 2 default strategies, the model has no clinical utility for the misclassification costs associated with *t*. Net Benefit is recommended by the TRIPOD reporting guideline for clinical prediction modeling studies.^23^

### Monte Carlo simulation study

We used the ADEMP (aim, data, estimands, methods, performance) guideline to design and report the simulation study.^24^

#### Aim

The aim of this study was to investigate the impact of class imbalance corrections on model performance in terms of discrimination, calibration, and classification.

#### Data generating mechanism

Twenty-four scenarios were investigated by varying the following simulation factors: original training set size (*N*) (2500 or 5000), number of predictors (*p*) (3, 6, 12, or 24), and outcome event fraction (0.3, 0.1, 0.01). The values for *p* and the event fraction reflect common situations for clinical prediction models.^25^ A sample size of 2500 will include 25 events on average when the event fraction is 1%. Smaller values for *N* may hence lead to computational issues. Candidate predictor variables were drawn from a multivariate standard normal distribution with zero correlation between predictors. Then, the outcome probability of each case was computed by applying a logistic function to the generated predictors. The coefficients of this function were approximated numerically for each scenario (see Supplementary Material), such that the predictors were of equal strength, the c-statistic of the data generating model was approximately 0.75, and the outcome prevalence expected in accordance with the simulation condition. The outcome variable was sampled from a binomial distribution.

#### Estimands/targets of analysis

The focus is on discrimination, calibration, and classification performance of the fitted models on a large out-of-sample dataset.

#### Methods

For each generated training dataset, 4 prediction model training datasets were created: *D*_uncorrected_, *D*_RUS_, *D*_ROS_, and *D*_SMOTE_. On each of these datasets, SLR and Ridge models were fit. This resulted in 8 different prediction models per simulation scenario. Because we anticipated imbalance correction would lead to overestimation of probabilities (ie, that the model intercept would be too high), we also implemented a logistic recalibration approach for the models developed on *D*_RUS_, *D*_ROS_, and *D*_SMOTE_, resulting in another 6 models.^26^ This recalibration was done by fitting a logistic regression model on the training dataset with the logit of the estimated probabilities from the initial model as an offset variable and the intercept as the only free parameter:

$$\log\left(\frac{\pi_{i,\mathrm{recalibrated}}}{{1-\pi }_{i,\;\mathrm{recalibrated}}}\right)=a+\log\left(\frac{{\hat{\pi }}_{i}}{1-{\hat{\pi }}_{i}}\right),$$

For each scenario, 2,000 simulation runs were performed. In each run, a newly simulated training dataset was used. To evaluate the performance of the resulting models for a given scenario, a single test set per scenario was simulated with size *N *=* *100,000 using the same data generating mechanism.

#### Performance metrics

We applied each model on its respective test set, and calculated the AUROC, accuracy, sensitivity, specificity, calibration intercept, and slope. To convert the estimated probabilities into a dichotomous prediction, a default risk threshold of 0.5 was used. For models trained on uncorrected training datasets, we also used a threshold that is equal to the true event fraction. The primary metric was the calibration intercept.^21^^,^^22^

#### Software and error handling

All analyses were performed using R version 3.6.2 (www.R-project.org). The simulation study was performed on a high-performance computing facility running on a Linux-based Operating System (CentOS7). To fit the regression models, the R packages stat and glmnet version 4.0-2 were used.^27^^,^^28^ To implement SMOTE and simulate data from a multivariate normal distribution, we respectively used the smotefamily (Siriseriwan W. *smotefamily: A Collection of Oversampling Techniques for Class Imbalance Problem Based on SMOTE*. 2019. R package version 1.3.1) and the MASS version 7.3-51.5 R packages.^27^ The code is available via https://github.com/benvancalster/classimb_calibration.

Errors in the generation of the training datasets and estimation of the models were closely monitored (details in Supplementary Material).^29^ A summary of the datasets in which data separation occurred is given in in Supplementary Table S1.

## RESULTS

### Case study

**Figure 2.**
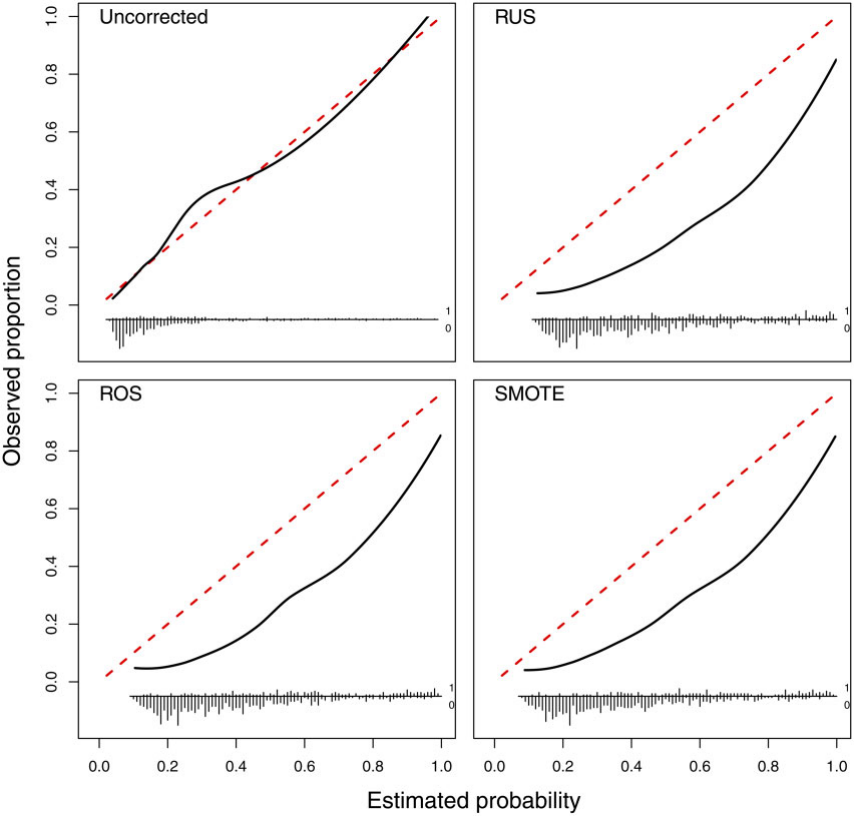
Flexible calibration curves on the test set for the Ridge models to diagnose ovarian cancer.

There was little variation in discrimination across algorithms and imbalance correction methods, with average AUROC of 0.79 to 0.80 (Supplementary Table S2). The calibration curves indicate that all imbalance correction methods had strong impact on calibration, yielding strongly overestimated probability estimates after imbalance correction but not without correction (Figure 2). This is confirmed by the calibration intercepts: these were 0.06 (95% CI −0.16 to 0.26) for SLR and 0.05 (−0.16 to 0.26) for Ridge on training data without imbalance correction, but varied between −1.32 (−1.54 to −1.11; SMOTE followed by SLR) and −1.50 (−1.72 to −1.28; RUS followed by SLR) when using imbalance corrections. The calibration slope was closest to the target value of 1 for models based on uncorrected data (0.99 for SLR, 1.03 for Ridge) and lowest (ie, worst) for models after RUS (0.85 for SLR, 0.93 for Ridge). When using the 0.5 probability threshold on models trained on uncorrected data, specificity (96% for SLR and Ridge) was clearly higher than sensitivity (31% for SLR, 29% for Ridge). As expected, sensitivity increased and specificity decreased by changing the classification threshold for models based on uncorrected data or using the 0.5 threshold for models after imbalance correction (sensitivities between 69% and 75%, specificities between 74% and 78%).

**Figure 3.**
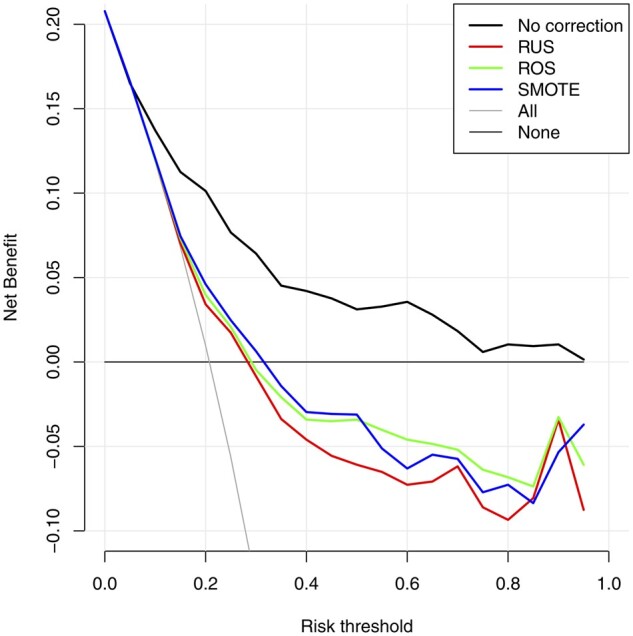
Decision curves on the test for the Ridge models to diagnose ovarian cancer. “All” refers to classifying all individuals as high risk (and hence treat all), “None” refers to classifying all individuals as low risk (and hence treat none).

Our results also show that the overestimation of the probability of a malignancy for models that were trained on imbalance corrected datasets could lead to overtreatment: too many individuals would exceed a given probability threshold and would be selected for treatment (for instance, referral to specialized gynecologic oncology centers for surgery). This is reflected in the Net Benefit measurement of clinical utility (Figure 3). The decision curves show that models trained on imbalance corrected datasets had strongly reduced clinical utility, even to the extent that the Net Benefit was negative when using a probability threshold of 0.3 or higher to select individuals for treatment.

### Simulation study

**Figure 4.**
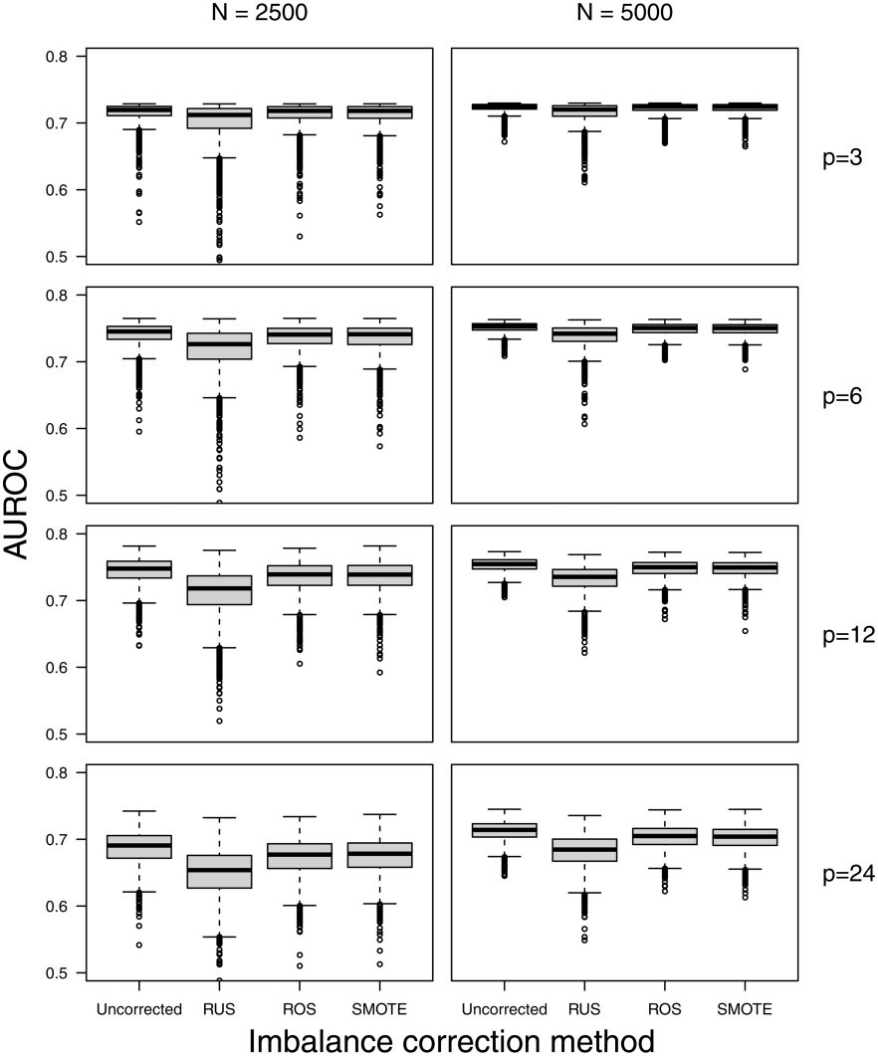
Test set AUROC for the Ridge models in the simulation scenarios with an event fraction of 1%.

We show results for Ridge models in scenarios with a 1% event fraction in the main text, and show other results in Supplementary Material. The simulation results did not provide evidence that imbalance correction methods systematically improved the AUROC compared to developing models on the original (imbalanced) training data (Figure 4; Supplementary Figures S1–S5 and Table S3). The median AUROC of models trained on uncorrected data was never lower than the median AUROC of models after RUS, ROS, or SMOTE. For RUS, the median AUROC was often lower, with larger differences when event fraction was lower, training set size was lower, and number of predictors was higher.

**Figure 5.**
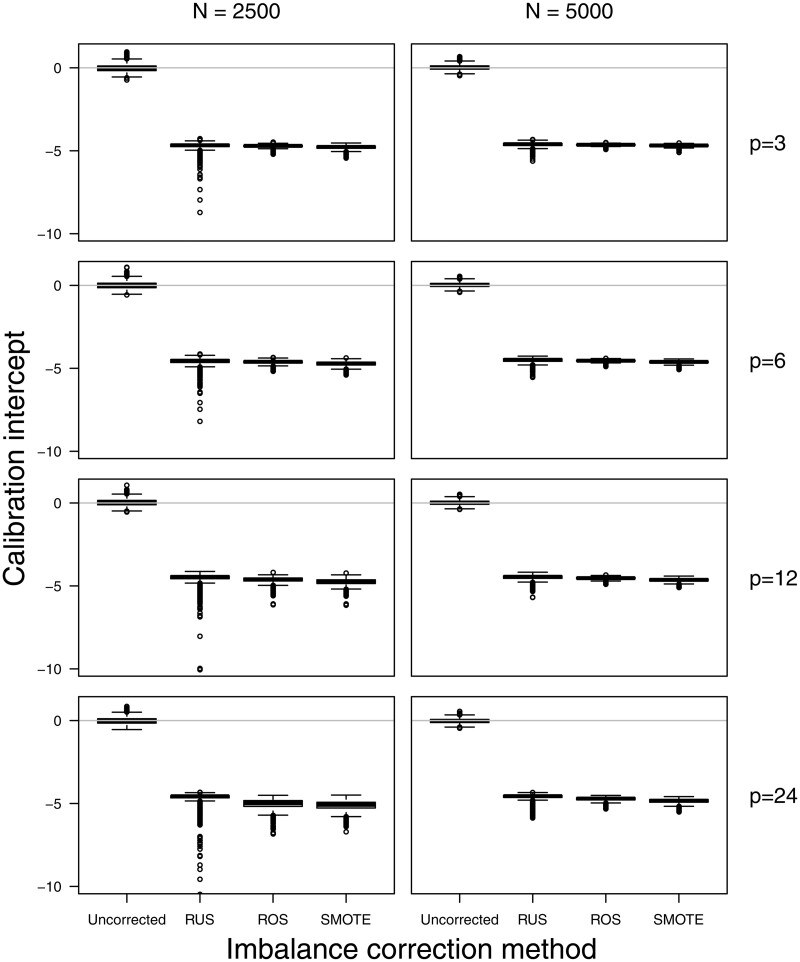
Test set calibration intercept for the Ridge models in the simulation scenarios with an event fraction of 1%.

Training models on imbalance corrected datasets resulted in severe overestimation of the estimated probabilities as evidenced by the negative calibration intercepts (Figure 5; Supplementary Figures S6–S10 and Table S4). Models trained on uncorrected data had median calibration intercepts between −0.05 and 0.03. Imbalance correction methods had median calibration intercepts of −4.5 or lower for scenarios with a 1% event fraction, −2.1 or lower for scenarios with a 10% event fraction, and −0.7 or lower for scenarios with a 30% event fraction. This was corrected by applying the recalibration procedure. Using the original (imbalanced) data: recalibration improved median calibration intercepts to values between −0.07 and 0.03 (Supplementary Figures S11–S16 and Table S5). One exception involved the training of SLR models after RUS in the scenario with 1% event fraction, a training set size of 2500, and 24 predictors. Using RUS implied that the model with 24 predictors was trained on a dataset including only 25 events and 25 nonevents on average, leading to lack of convergence of the SLR model (separation, see Supplementary Table S1), but not for the Ridge model.

**Figure 6.**
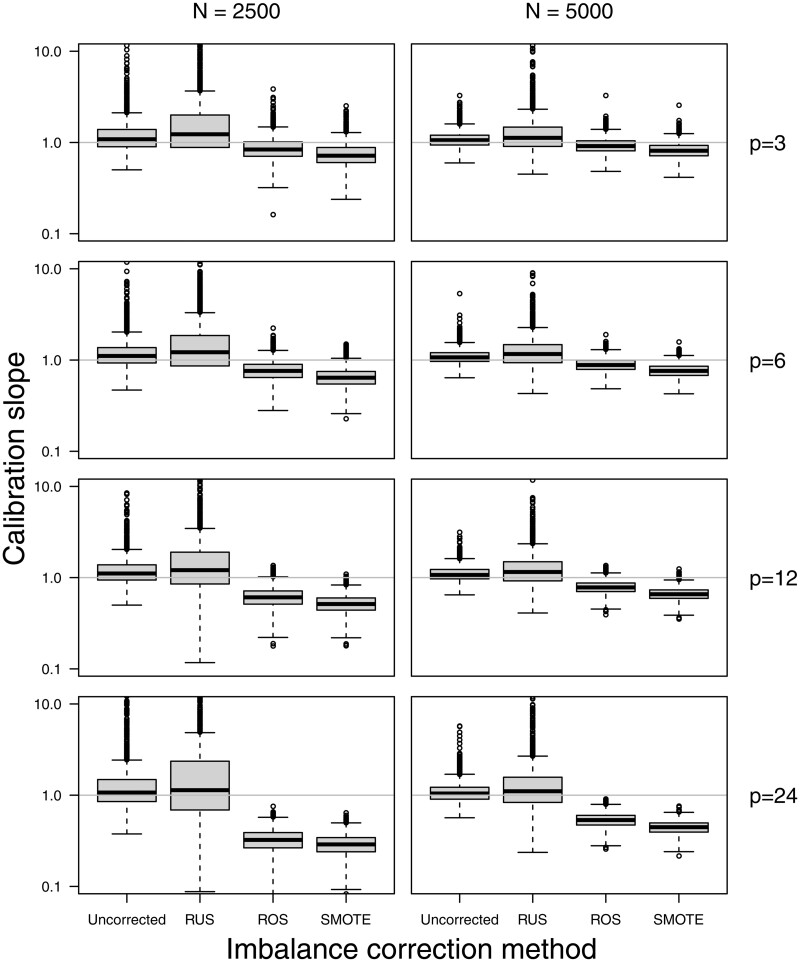
Test set calibration slope for the Ridge models in the simulation scenarios with an event fraction of 1%.

The use of SMOTE, and to a lesser extent ROS, resulted probability estimates that were too extreme as evidenced by median calibration slopes below 1 both for Ridge and SLR models (Figure 6; Supplementary Figures S17–S21 and Table S6). The use of RUS resulted in good median calibration slopes yet with high variability for Ridge models, and in slopes that were often well below 1 for SLR models. These findings for the calibration slope were more evident for lower event fraction, lower training set size, and a larger number of predictors. Median calibration slopes below 1 were also observed for SLR models developed on uncorrected training data, however these median slopes where still higher than those for models developed after RUS, ROS, or SMOTE were still lower.

Regarding classification, using a probability threshold of 0.5 for models trained on uncorrected data resulted in median sensitivities of 0% and median specificities of 100% when the true event fraction was 1% (Supplementary Figures S22–S39 and Tables S7 and S8). More balanced results for sensitivity and specificity were obtained by either using imbalance correction methods or shifting the probability threshold (Supplementary Figures S29–S40).

## DISCUSSION

The key finding of our work is that training logistic regression models on imbalance corrected data did not lead to better AUROC compared to models trained on uncorrected data, but did result in strong and systematic overestimation of the probability for the minority class. In addition, all imbalance corrections had negative consequences for the calibration slope. The lower the event fraction, the more outspoken the results.

Strong miscalibration reduces the clinical utility of a prediction model.^30^ Models yielding probability estimates that are clearly too high may lead to overtreatment. For example, if a model overestimates the risk of malignancy of a detected ovarian tumor, the decision to refer patients to specialized surgery may be taken too quickly. Class imbalance is often framed as problematic in the context of prediction models that classify patients into low-risk versus high-risk groups.^1–3^^,^^31^ Nevertheless, for clinical prediction models the accurate estimation of probabilities is essential to help in defining such low-risk and high-risk groups. For instance, clinical staff using the model to support treatment decisions may choose probability thresholds to match the assumed misclassification costs that best fit the context. The context is defined by issues such as the healthcare system, other relevant patient information, and patient values.^12^ Hence, when probability estimation is important, calibration becomes a central performance criterion.^32^^,^^33^ Although we realize that prediction models cannot replace clinicians, and that clinicians should not blindly follow a prediction model, research suggests that incorrect model predictions lead to inferior clinical decisions.^34^

The relation between correction for class imbalance and calibration of estimated probabilities is rarely made. For instance, it is not discussed in some key publications on class imbalance for prediction models.^1–3^^,^^31^ A study from 2011 hinted at this link by stating that “the predicted probability using a logistic regression model is closest to the true probability when the sample has the same class distribution as the original population,” and that differences in class distribution between study sample and population should be avoided.^35^ However, the authors did not systematically study typical imbalance correction methods, and the simulations were based on an unrealistic setting with only 1 predictor and a true AUROC around 0.99. Another study into imbalance corrections quantified calibration incorrectly by using Brier score and class-specific Brier scores.^36^ Brier score is a statistically proper measure of overall measure of performance, that captures both discrimination and calibration. This study incorrectly claimed that using RUS improved probability estimates compared to using uncorrected data in the minority class based on observed lower values of the Brier score in the minority class. This, however, does not mean that the probability estimates are well calibrated, but simply means that the probabilities in the minority class are closer to 1. This is consistent with our findings: probability estimates under RUS are indeed miscalibrated toward too extreme values.

Another study did indicate that undersampling distorts probability estimates and increases the variance of the prediction model (which relates to the higher tendency of overfitting due to artificially reducing sample size).^37^ However, the study focused on classification accuracy, by claiming that the effect of undersampling on accuracy depends on many factors such that it is difficult to know when it will lead to better accuracy. In contrast, our study suggests that, at least for logistic regression models, RUS (or ROS or SMOTE) is unlikely to lead to better discrimination or separability between the minority and majority classes.

It is well known that for developing robust clinical prediction models, the sample size should be large enough to reduce overfitting.^13–15^^,^^17^^,^^38^ Recent studies indicate that the most important factors to determine overall sample size are the event fraction, the number of considered parameters, and the expected performance of the model. From that perspective, undersampling is a very counterintuitive approach, because it deliberately decreases sample size available for model training, which may lead to an artificially increased risk of overfitting. The stronger the imbalance, the more undersampling may induce overfitting. Our results are consistent with this expectation: RUS resulted in lower AUROC values on the test data.

Based on the results presented in this study, it is warranted to conduct follow-up studies that systematically study the impact of imbalance corrections on discrimination and calibration performance, in particular in the context of other algorithms and more severe imbalance levels than what was studied here. For instance, the calibration performance of increasingly popular approaches for prediction model development such as Random Forest, Support Vector Machines and Neural Networks remains to be investigated. Also, other imbalance correction methods exist, such as weighting, cost-sensitive learning, or variants of RUS, ROS, and SMOTE.^3^^,^^31^^,^^39^ We did not include scenarios with event fraction <1%, so more detailed assessments of situations with more severe imbalance may be insightful. However, the observed problems are unlikely to suddenly disappear with even lower event fractions. We anticipate that risk miscalibration will remain present regardless of type of model or imbalance correction technique, unless the models are recalibrated. However, class imbalance correction followed by recalibration is only worth the effort if imbalance correction leads to better discrimination of the resulting models.

## CONCLUSION

Our study shows that correcting class imbalance did not result in better prediction models based on standard or ridge logistic regression. The imbalance corrections resulted in inaccurate probability estimates without improving discrimination in terms of AUROC. We therefore warn researchers for the limitations of imbalance corrections when developing a prediction model.

## FUNDING

BVC and DT are supported by the Research Foundation—Flanders (FWO) grant number G097322N and Internal Funds KU Leuven (grant number C24M/20/064).

## AUTHOR CONTRIBUTIONS

Conception and design: All authors. Data analysis: RvdG and BVC. Data interpretation: All authors. Drafting the manuscript: RvdG and BVC. Revising the manuscript: All authors. Approval of submitted version: All authors. Accountability of own contributions: All authors.

## SUPPLEMENTARY MATERIAL

Supplementary material is available at *Journal of the American Medical Informatics Association* online.

## CONFLICT OF INTEREST STATEMENT

The authors declare that they have no competing interests.

## Supplementary Material

ocac093_Supplementary_Data

## Data Availability

The ovarian tumor dataset cannot be shared for ethical/privacy reasons.
