# Supplementary material for: The harm of class imbalance corrections for risk prediction models: illustration and simulation using logistic regression
Source: J Am Med Inform Assoc. 2022 Jun 10;29(9):1525–34. doi: 10.1093/jamia/ocac093 (PMC9382395; doi:10.1093/jamia/ocac093)

The harm of class imbalance corrections for risk prediction models:

illustration and simulation using logistic regression

Ruben van den Goorbergh^1^, Maarten van Smeden^1^, Dirk Timmerman,^2,3^ Ben Van Calster B^2,4,5^

1 Julius Center for Health Sciences and Primary Care, UMC Utrecht, Utrecht University, Utrecht, Netherlands; 2 Department of Development and Regeneration, KU Leuven, Leuven, Belgium; 3 Department of Obstetrics and Gynecology, University Hospitals Leuven, Leuven, Belgium; 4 Department of Biomedical Data Sciences, Leiden University Medical Center, Leiden, Netherlands; 5 EPI-center, KU Leuven, Leuven, Belgium

**SUPPLEMENTARY MATERIAL**

**Logistic regression models**

Let $Y$ denote the outcome taking on the value 1 for events and value 0 for non-events, $y_{i}$ the observed value of $Y$ for the for a given individual *i*, $\mathbf{X}$ a vector of *p* predictor variables, $\mathbf{x}_{\mathbf{i}}$ the observed values for the predictors for a given individual *i*, and $\pi_{i}=P\left( Y=1|{\mathbf{X}=\mathbf{x}}_{i} \right)$ be the probability of an event for a given individual *i*. Assuming for simplicity only linear associations of the predictors and no interactions between predictors, logistic regression for $\pi_{i}$ can be defined as follows

$log\left( \frac{\pi_{i}}{{1-\pi}_{i}} \right)=\alpha+\mathbf{x}_{\mathbf{i}}\boldsymbol{\beta}$,

where $\alpha$ is the intercept and $\boldsymbol{\beta}$ a vector containing the regression coefficient for each predictor. Nonlinear effects of predictors (such as quadratic terms) and interaction terms between predictors can be incorporated by considering these terms as additional terms in $\mathbf{X}$. Using a dataset with $N$ individuals ($i = 1, ..., N$), the regression coefficients are estimated using maximum likelihood estimation, i.e. by maximizing the log-likelihood function

$l\left( \alpha,\boldsymbol{\beta} \right)=\sum_{i=1}^{N} \left\{ y_{i}log\left( \pi_{i}\left( \alpha,\boldsymbol{\beta} \right) \right)+\left( 1-y_{i} \right)log\left( 1-\pi_{i}\left( \alpha,\boldsymbol{\beta} \right) \right) \right\}$.

We will refer to this model as standard logistic regression (SLR).

Alternatively, when using a ridge penalty, the following penalized version of the log-likelihood function is used for estimating $\boldsymbol{\beta}$, in order to penalize the coefficients towards zero:

${l\left( \alpha,\boldsymbol{\beta} \right)}_{ridge}=l\left( \alpha,\boldsymbol{\beta} \right)-\lambda\sum_{j=1}^{p} \beta_{j}^{2}$.

We refer to this model simply as Ridge. Here, $\lambda$ is a hyperparameter that controls the amount of penalization. We tuned $\lambda$ using 10-fold cross-validation on the deviance from a grid of 251 possible values between 0 (no penalization) and 64 (very strong penalization). The non-null values in this grid were equidistant on logarithmic scale.

**True coefficient estimation**

The intercepts and coefficients that result in the desired true AUROC and event fraction were estimated by numerical optimization using the optim() function from the stats R package. The method used for optimization was the Broyden-Fletcher-Goldfarb-Shanno (BFGS) algorithm. The function to be minimized was the sum of the difference between the observed AUROC and the desired AUROC squared and the difference between the observed prevalence and the desired prevalence squared. As this method can lead to slightly varying results, the minimization procedure was deployed 20 times after which the median coefficient values were chosen. This process was repeated for 20 generated data sets of size *N* = 10^5^, after which again the median coefficient values of all 20 repetitions were chosen. The final coefficient values validated on independently generated data sets of size *N* = 10^5^.

**Error handling**

Errors in the generation of the development datasets were closely monitored, a table summarizing the error occurrence per simulation cell is included in the article. Data separation in the development datasets was assumed when the apparent AUROC in the development data set was equal to 1, based on the maximum likelihood logistic regression model. Because, in practice, clinical prediction modelers should not develop prediction models on separated data, separated data sets were removed from the analysis. Very few cases of data separation, or no cases at all, were expected in the generated development data sets, given that the true AUROC was set to be approximately 0.75 and the minimum sample size was 2,500. However, data separation was likely to occur when random undersampling is used.

If a development data set contained cases of only one class, this data set was excluded from the analysis; the simulation results are based on complete case analysis. Development datasets with fewer than 8 events or non-events can cause severe problems in estimating tuning parameter $\lambda$ in ridge logistic regression using 10-fold cross-validation. In such cases, leave-one-out cross-validation was used to estimate $\lambda$. When there were less than 6 minority-class events in the development data set, the SMOTE-algorithm fails when using the default setting because it searches for the *k=5* nearest neighbors. In such cases *k* was set to the number of minority-class events minus 1. In the most extreme scenario with event fraction 0.01 and sample size 2,500, the probability of generating a data set with < 8 events was only 0.00002 (1 in 50,000).

Table S1. Number and percentage of the 2000 runs with data separation for simulation scenarios with event fraction of 0.01. No issues were encountered for event fractions of 0.1 or 0.3.

|  |  | **Imbalance correction method** | | | |
| --- | --- | --- | --- | --- | --- |
| **N** | **p** | **Uncorrected** | **RUS** | **ROS** | **SMOTE** |
| 2500 | 3 | 0 | 0 | 0 | 0 |
| 2500 | 6 | 0 | 3 (0.2%) | 0 | 0 |
| 2500 | 12 | 0 | 52 (2.6%) | 0 | 0 |
| 2500 | 24 | 0 | 1238 (61.9%) | 0 | 0 |
| 5000 | 3 | 0 | 0 | 0 | 0 |
| 5000 | 6 | 0 | 0 | 0 | 0 |
| 5000 | 12 | 0 | 0 | 0 | 0 |
| 5000 | 24 | 0 | 9 (0.5%) | 0 | 0 |

Table S2. Test set performance for the SLR and Ridge models to diagnose ovarian malignancy.

|  | **Uncorrected** | **RUS** | **ROS** | **SMOTE** |
| --- | --- | --- | --- | --- |
| *SLR model* | | | | |
| AUC  (95% CI) | 0.79  (0.75; 0.83) | 0.79  (0.75; 0.83) | 0.80  (0.75; 0.84) | 0.79  (0.75; 0.83) |
| Accuracy, t=0.5 | 82 | 73 | 76 | 76 |
| Sensitivity, t=0.5 | 31 | 71 | 70 | 69 |
| Specificity, t=0.5 | 96 | 74 | 77 | 78 |
| Accuracy, t=0.192 | 75 | Nc | Nc | Nc |
| Sensitivity, t=0.192 | 71 | Nc | Nc | Nc |
| Specificity, t=0.192 | 76 | Nc | Nc | Nc |
| Calibration intercept  (95% CI) | 0.06  (-0.16; 0.26) | -1.50  (-1.72; -1.28) | -1.37  (-1.59; -1.16) | -1.32  (-1.54; -1.11) |
| Calibration slope  (95% CI) | 0.99  (0.81; 1.20) | 0.85  (0.68; 1.02) | 0.93  (0.76; 1.12) | 0.92  (0.75; 1.11) |
| *Ridge model* | | | | |
| AUC  (95% CI) | 0.79  (0.75; 0.83) | 0.80  (0.75; 0.83) | 0.80  (0.75; 0.84) | 0.79  (0.75; 0.83) |
| Accuracy, t=0.5 | 82 | 73 | 76 | 76 |
| Sensitivity, t=0.5 | 29 | 71 | 70 | 69 |
| Specificity, t=0.5 | 96 | 74 | 77 | 78 |
| Accuracy, t=0.192 | 75 | Nc | Nc | Nc |
| Sensitivity, t=0.192 | 70 | Nc | Nc | Nc |
| Specificity, t=0.192 | 76 | Nc | Nc | Nc |
| Calibration intercept  (95% CI) | 0.05  (-0.16; 0.26) | -1.48  (-1.70; -1.27) | -1.38  (-1.60; -1.17) | -1.33  (-1.55; -1.12) |
| Calibration slope  (95% CI) | 1.03  (0.84; 1.25) | 0.93  (0.76; 1.12) | 0.95  (0.77; 1.14) | 0.94  (0.77; 1.13) |

Table S3. Test set AUROC, reported as median (IQR) over the 2000 simulation runs for SLR and Ridge in the 24 simulation scenarios.

|  |  |  |  | **SLR** | | | | **RIDGE** | | | |
| --- | --- | --- | --- | --- | --- | --- | --- | --- | --- | --- | --- |
| **Scenario** | **EF** | **N** | **p** | **Uncorrected** | **RUS** | **ROS** | **SMOTE** | **Uncorrected** | **RUS** | **ROS** | **SMOTE** |
| 1 | 0.01 | 2500 | 3 | 0.72 (0.71;0.72) | 0.71 (0.69;0.72) | 0.72 (0.71;0.72) | 0.72 (0.71;0.72) | 0.72 (0.71;0.72) | 0.71 (0.69;0.72) | 0.72 (0.71;0.72) | 0.72 (0.71;0.72) |
| 2 | 0.01 | 2500 | 6 | 0.75 (0.73;0.75) | 0.72 (0.69;0.74) | 0.74 (0.73;0.75) | 0.74 (0.73;0.75) | 0.75 (0.73;0.75) | 0.73 (0.70;0.74) | 0.74 (0.73;0.75) | 0.74 (0.73;0.75) |
| 3 | 0.01 | 2500 | 12 | 0.75 (0.73;0.76) | 0.70 (0.67;0.72) | 0.74 (0.72;0.75) | 0.74 (0.72;0.75) | 0.75 (0.73;0.76) | 0.72 (0.69;0.74) | 0.74 (0.72;0.75) | 0.74 (0.72;0.75) |
| 4 | 0.01 | 2500 | 24 | 0.69 (0.67;0.70) | 0.59 (0.56;0.62) | 0.68 (0.66;0.69) | 0.68 (0.66;0.69) | 0.69 (0.67;0.71) | 0.65 (0.63;0.68) | 0.68 (0.66;0.69) | 0.68 (0.66;0.69) |
| 5 | 0.01 | 5000 | 3 | 0.73 (0.72;0.73) | 0.72 (0.71;0.73) | 0.72 (0.72;0.73) | 0.72 (0.72;0.73) | 0.73 (0.72;0.73) | 0.72 (0.71;0.73) | 0.72 (0.72;0.73) | 0.72 (0.72;0.73) |
| 6 | 0.01 | 5000 | 6 | 0.75 (0.75;0.76) | 0.74 (0.73;0.75) | 0.75 (0.74;0.76) | 0.75 (0.74;0.76) | 0.75 (0.75;0.76) | 0.74 (0.73;0.75) | 0.75 (0.74;0.76) | 0.75 (0.74;0.76) |
| 7 | 0.01 | 5000 | 12 | 0.75 (0.75;0.76) | 0.73 (0.71;0.74) | 0.75 (0.74;0.76) | 0.75 (0.74;0.76) | 0.75 (0.75;0.76) | 0.74 (0.72;0.75) | 0.75 (0.74;0.76) | 0.75 (0.74;0.76) |
| 8 | 0.01 | 5000 | 24 | 0.71 (0.70;0.72) | 0.67 (0.64;0.69) | 0.70 (0.69;0.72) | 0.70 (0.69;0.71) | 0.71 (0.70;0.72) | 0.68 (0.67;0.70) | 0.70 (0.69;0.72) | 0.70 (0.69;0.71) |
| 9 | 0.1 | 2500 | 3 | 0.73 (0.73;0.73) | 0.73 (0.73;0.73) | 0.73 (0.73;0.73) | 0.73 (0.73;0.73) | 0.73 (0.73;0.73) | 0.73 (0.73;0.73) | 0.73 (0.73;0.73) | 0.73 (0.73;0.73) |
| 10 | 0.1 | 2500 | 6 | 0.74 (0.74;0.74) | 0.74 (0.74;0.74) | 0.74 (0.74;0.74) | 0.74 (0.74;0.74) | 0.74 (0.74;0.74) | 0.74 (0.74;0.74) | 0.74 (0.74;0.74) | 0.74 (0.74;0.74) |
| 11 | 0.1 | 2500 | 12 | 0.75 (0.75;0.75) | 0.75 (0.74;0.75) | 0.75 (0.75;0.75) | 0.75 (0.75;0.75) | 0.75 (0.75;0.75) | 0.75 (0.74;0.75) | 0.75 (0.75;0.75) | 0.75 (0.75;0.75) |
| 12 | 0.1 | 2500 | 24 | 0.73 (0.73;0.73) | 0.72 (0.71;0.72) | 0.73 (0.72;0.73) | 0.73 (0.72;0.73) | 0.73 (0.73;0.73) | 0.72 (0.71;0.72) | 0.73 (0.72;0.73) | 0.73 (0.72;0.73) |
| 13 | 0.1 | 5000 | 3 | 0.73 (0.73;0.73) | 0.73 (0.73;0.73) | 0.73 (0.73;0.73) | 0.73 (0.73;0.73) | 0.73 (0.73;0.73) | 0.73 (0.73;0.73) | 0.73 (0.73;0.73) | 0.73 (0.73;0.73) |
| 14 | 0.1 | 5000 | 6 | 0.75 (0.75;0.75) | 0.74 (0.74;0.75) | 0.75 (0.74;0.75) | 0.75 (0.74;0.75) | 0.75 (0.75;0.75) | 0.74 (0.74;0.75) | 0.75 (0.74;0.75) | 0.75 (0.74;0.75) |
| 15 | 0.1 | 5000 | 12 | 0.75 (0.75;0.75) | 0.75 (0.75;0.75) | 0.75 (0.75;0.75) | 0.75 (0.75;0.75) | 0.75 (0.75;0.75) | 0.75 (0.75;0.75) | 0.75 (0.75;0.75) | 0.75 (0.75;0.75) |
| 16 | 0.1 | 5000 | 24 | 0.74 (0.74;0.74) | 0.73 (0.73;0.73) | 0.74 (0.74;0.74) | 0.74 (0.74;0.74) | 0.74 (0.74;0.74) | 0.73 (0.73;0.74) | 0.74 (0.74;0.74) | 0.74 (0.74;0.74) |
| 17 | 0.3 | 2500 | 3 | 0.74 (0.74;0.74) | 0.74 (0.74;0.74) | 0.74 (0.74;0.74) | 0.74 (0.74;0.74) | 0.74 (0.74;0.74) | 0.74 (0.74;0.74) | 0.74 (0.74;0.74) | 0.74 (0.74;0.74) |
| 18 | 0.3 | 2500 | 6 | 0.74 (0.74;0.74) | 0.74 (0.74;0.74) | 0.74 (0.74;0.74) | 0.74 (0.74;0.74) | 0.74 (0.74;0.74) | 0.74 (0.74;0.74) | 0.74 (0.74;0.74) | 0.74 (0.74;0.74) |
| 19 | 0.3 | 2500 | 12 | 0.75 (0.74;0.75) | 0.74 (0.74;0.75) | 0.75 (0.74;0.75) | 0.75 (0.74;0.75) | 0.75 (0.74;0.75) | 0.74 (0.74;0.75) | 0.75 (0.74;0.75) | 0.75 (0.74;0.75) |
| 20 | 0.3 | 2500 | 24 | 0.74 (0.73;0.74) | 0.73 (0.73;0.73) | 0.73 (0.73;0.74) | 0.73 (0.73;0.74) | 0.74 (0.73;0.74) | 0.73 (0.73;0.73) | 0.73 (0.73;0.74) | 0.73 (0.73;0.74) |
| 21 | 0.3 | 5000 | 3 | 0.74 (0.74;0.74) | 0.74 (0.74;0.74) | 0.74 (0.74;0.74) | 0.74 (0.74;0.74) | 0.74 (0.74;0.74) | 0.74 (0.74;0.74) | 0.74 (0.74;0.74) | 0.74 (0.74;0.74) |
| 22 | 0.3 | 5000 | 6 | 0.74 (0.74;0.74) | 0.74 (0.74;0.74) | 0.74 (0.74;0.74) | 0.74 (0.74;0.74) | 0.74 (0.74;0.74) | 0.74 (0.74;0.74) | 0.74 (0.74;0.74) | 0.74 (0.74;0.74) |
| 23 | 0.3 | 5000 | 12 | 0.75 (0.75;0.75) | 0.75 (0.75;0.75) | 0.75 (0.75;0.75) | 0.75 (0.75;0.75) | 0.75 (0.75;0.75) | 0.75 (0.75;0.75) | 0.75 (0.75;0.75) | 0.75 (0.75;0.75) |
| 24 | 0.3 | 5000 | 24 | 0.74 (0.74;0.74) | 0.74 (0.74;0.74) | 0.74 (0.74;0.74) | 0.74 (0.74;0.74) | 0.74 (0.74;0.74) | 0.74 (0.74;0.74) | 0.74 (0.74;0.74) | 0.74 (0.74;0.74) |

Table S4. Test set calibration intercept, reported as median (IQR) over the simulation 2000 runs for SLR and Ridge in the 24 simulation scenarios.

|  |  |  |  | **SLR** | | | | **RIDGE** | | | |  |
| --- | --- | --- | --- | --- | --- | --- | --- | --- | --- | --- | --- | --- |
| **Scen.** | **EF** | **N** | **p** | **Uncorrected** | **RUS** | **ROS** | **SMOTE** | **Uncorrected** | **RUS** | **ROS** | **SMOTE** | |
| 1 | 0.01 | 2500 | 3 | -0.04 (-0.17;0.11) | -4.84 (-5.10;-4.69) | -4.71 (-4.76;-4.67) | -4.78 (-4.85;-4.72) | -0.04 (-0.17;0.11) | -4.67 (-4.75;-4.60) | -4.70 (-4.75;-4.67) | -4.77 (-4.85;-4.71) | |
| 2 | 0.01 | 2500 | 6 | -0.01 (-0.14;0.13) | -5.01 (-5.46;-4.74) | -4.61 (-4.68;-4.56) | -4.71 (-4.81;-4.64) | -0.01 (-0.14;0.13) | -4.54 (-4.65;-4.47) | -4.61 (-4.67;-4.55) | -4.70 (-4.80;-4.63) | |
| 3 | 0.01 | 2500 | 12 | 0.02 (-0.11;0.15) | -5.92 (-7.08;-5.25) | -4.62 (-4.73;-4.54) | -4.76 (-4.89;-4.65) | 0.02 (-0.11;0.15) | -4.46 (-4.57;-4.39) | -4.60 (-4.70;-4.53) | -4.73 (-4.85;-4.63) | |
| 4 | 0.01 | 2500 | 24 | -0.05 (-0.18;0.09) | <-100 (<-100;-14.4) | -5.04 (-5.25;-4.87) | -5.15 (-5.36;-4.99) | -0.02 (-0.15;0.11) | -4.56 (-4.66;-4.53) | -4.98 (-5.18;-4.82) | -5.08 (-5.27;-4.93) | |
| 5 | 0.01 | 5000 | 3 | 0.02 (-0.08;0.12) | -4.71 (-4.84;-4.61) | -4.64 (-4.67;-4.62) | -4.68 (-4.73;-4.65) | 0.02 (-0.08;0.12) | -4.60 (-4.68;-4.55) | -4.64 (-4.67;-4.62) | -4.68 (-4.72;-4.65) | |
| 6 | 0.01 | 5000 | 6 | 0.03 (-0.06;0.12) | -4.69 (-4.87;-4.56) | -4.54 (-4.58;-4.51) | -4.62 (-4.67;-4.57) | 0.03 (-0.06;0.12) | -4.49 (-4.57;-4.43) | -4.54 (-4.57;-4.51) | -4.61 (-4.67;-4.57) | |
| 7 | 0.01 | 5000 | 12 | 0.01 (-0.08;0.11) | -4.93 (-5.20;-4.73) | -4.54 (-4.58;-4.49) | -4.65 (-4.71;-4.58) | 0.01 (-0.08;0.11) | -4.45 (-4.55;-4.40) | -4.53 (-4.58;-4.49) | -4.64 (-4.70;-4.58) | |
| 8 | 0.01 | 5000 | 24 | -0.03 (-0.13;0.07) | -6.10 (-6.89;-5.58) | -4.72 (-4.80;-4.66) | -4.85 (-4.95;-4.78) | -0.02 (-0.12;0.07) | -4.56 (-4.63;-4.51) | -4.71 (-4.78;-4.65) | -4.83 (-4.92;-4.76) | |
| 9 | 0.1 | 2500 | 3 | 0.01 (-0.04;0.05) | -2.20 (-2.23;-2.16) | -2.19 (-2.21;-2.18) | -2.15 (-2.18;-2.12) | 0.01 (-0.04;0.05) | -2.18 (-2.21;-2.15) | -2.19 (-2.20;-2.17) | -2.14 (-2.17;-2.11) | |
| 10 | 0.1 | 2500 | 6 | 0.00 (-0.05;0.04) | -2.17 (-2.21;-2.14) | -2.15 (-2.17;-2.14) | -2.12 (-2.15;-2.08) | 0.00 (-0.05;0.04) | -2.14 (-2.18;-2.11) | -2.15 (-2.16;-2.13) | -2.11 (-2.14;-2.08) | |
| 11 | 0.1 | 2500 | 12 | 0.00 (-0.04;0.05) | -2.16 (-2.20;-2.12) | -2.12 (-2.14;-2.10) | -2.09 (-2.13;-2.06) | 0.00 (-0.04;0.05) | -2.10 (-2.14;-2.07) | -2.11 (-2.13;-2.10) | -2.09 (-2.12;-2.05) | |
| 12 | 0.1 | 2500 | 24 | -0.02 (-0.06;0.03) | -2.26 (-2.31;-2.22) | -2.17 (-2.19;-2.15) | -2.15 (-2.19;-2.12) | -0.02 (-0.06;0.03) | -2.15 (-2.18;-2.12) | -2.16 (-2.17;-2.14) | -2.14 (-2.17;-2.10) | |
| 13 | 0.1 | 5000 | 3 | 0.00 (-0.03;0.03) | -2.20 (-2.23;-2.18) | -2.20 (-2.21;-2.19) | -2.15 (-2.18;-2.12) | 0.00 (-0.03;0.03) | -2.19 (-2.22;-2.17) | -2.20 (-2.21;-2.19) | -2.15 (-2.18;-2.11) | |
| 14 | 0.1 | 5000 | 6 | 0.01 (-0.02;0.05) | -2.15 (-2.18;-2.12) | -2.14 (-2.15;-2.13) | -2.10 (-2.13;-2.07) | 0.01 (-0.02;0.05) | -2.13 (-2.16;-2.11) | -2.14 (-2.15;-2.12) | -2.10 (-2.13;-2.07) | |
| 15 | 0.1 | 5000 | 12 | 0.00 (-0.03;0.03) | -2.14 (-2.17;-2.11) | -2.12 (-2.13;-2.11) | -2.09 (-2.13;-2.06) | 0.00 (-0.03;0.03) | -2.11 (-2.14;-2.09) | -2.12 (-2.13;-2.11) | -2.09 (-2.12;-2.05) | |
| 16 | 0.1 | 5000 | 24 | 0.00 (-0.03;0.03) | -2.20 (-2.23;-2.17) | -2.15 (-2.17;-2.14) | -2.13 (-2.16;-2.10) | 0.00 (-0.03;0.03) | -2.14 (-2.17;-2.12) | -2.15 (-2.16;-2.14) | -2.12 (-2.15;-2.09) | |
| 17 | 0.3 | 2500 | 3 | 0.01 (-0.03;0.04) | -0.84 (-0.85;-0.82) | -0.84 (-0.85;-0.82) | -0.69 (-0.72;-0.66) | 0.01 (-0.03;0.04) | -0.84 (-0.85;-0.82) | -0.84 (-0.85;-0.82) | -0.69 (-0.72;-0.66) | |
| 18 | 0.3 | 2500 | 6 | 0.00 (-0.03;0.03) | -0.84 (-0.86;-0.83) | -0.84 (-0.85;-0.82) | -0.70 (-0.73;-0.67) | 0.00 (-0.03;0.03) | -0.84 (-0.85;-0.82) | -0.84 (-0.85;-0.82) | -0.70 (-0.73;-0.66) | |
| 19 | 0.3 | 2500 | 12 | 0.00 (-0.04;0.03) | -0.83 (-0.85;-0.81) | -0.83 (-0.84;-0.81) | -0.70 (-0.73;-0.67) | 0.00 (-0.04;0.03) | -0.82 (-0.84;-0.81) | -0.82 (-0.84;-0.81) | -0.70 (-0.73;-0.66) | |
| 20 | 0.3 | 2500 | 24 | 0.01 (-0.03;0.04) | -0.84 (-0.86;-0.83) | -0.83 (-0.84;-0.81) | -0.69 (-0.73;-0.66) | 0.01 (-0.03;0.04) | -0.83 (-0.84;-0.81) | -0.82 (-0.83;-0.81) | -0.69 (-0.72;-0.65) | |
| 21 | 0.3 | 5000 | 3 | 0.00 (-0.02;0.02) | -0.84 (-0.85;-0.83) | -0.84 (-0.85;-0.83) | -0.70 (-0.72;-0.67) | 0.00 (-0.02;0.02) | -0.84 (-0.85;-0.83) | -0.84 (-0.85;-0.83) | -0.69 (-0.72;-0.67) | |
| 22 | 0.3 | 5000 | 6 | 0.00 (-0.02;0.03) | -0.83 (-0.84;-0.82) | -0.83 (-0.84;-0.82) | -0.69 (-0.71;-0.67) | 0.00 (-0.02;0.03) | -0.83 (-0.84;-0.82) | -0.83 (-0.84;-0.82) | -0.69 (-0.71;-0.67) | |
| 23 | 0.3 | 5000 | 12 | 0.00 (-0.02;0.02) | -0.83 (-0.84;-0.81) | -0.82 (-0.83;-0.81) | -0.70 (-0.72;-0.67) | 0.00 (-0.02;0.02) | -0.82 (-0.83;-0.81) | -0.82 (-0.83;-0.81) | -0.69 (-0.72;-0.67) | |
| 24 | 0.3 | 5000 | 24 | -0.01 (-0.03;0.01) | -0.85 (-0.86;-0.84) | -0.84 (-0.85;-0.83) | -0.71 (-0.73;-0.68) | -0.01 (-0.03;0.01) | -0.84 (-0.85;-0.83) | -0.84 (-0.85;-0.83) | -0.70 (-0.73;-0.68) | |

Table S5. Test set calibration intercept after recalibration for RUS/ROS/SMOTE, reported as median (IQR) over the 2000 runs for SLR and Ridge in the 24 simulation scenarios.

|  |  |  |  | **SLR** | | | **RIDGE** | | | |
| --- | --- | --- | --- | --- | --- | --- | --- | --- | --- | --- |
| **Scenario** | **EF** | **N** | **p** | **RUS** | **ROS** | **SMOTE** | **RUS** | **ROS** | **SMOTE** |  |
| 1 | 0.01 | 2500 | 3 | -0.04 (-0.17;0.11) | -0.04 (-0.17;0.11) | -0.04 (-0.18;0.11) | -0.04 (-0.17;0.11) | -0.04 (-0.17;0.11) | -0.04 (-0.18;0.11) |  |
| 2 | 0.01 | 2500 | 6 | 0.00 (-0.14;0.13) | -0.01 (-0.14;0.13) | -0.02 (-0.15;0.14) | 0.00 (-0.14;0.13) | -0.01 (-0.14;0.13) | -0.02 (-0.15;0.14) |  |
| 3 | 0.01 | 2500 | 12 | 0.02 (-0.11;0.16) | 0.01 (-0.12;0.16) | 0.01 (-0.13;0.16) | 0.02 (-0.11;0.16) | 0.01 (-0.12;0.16) | 0.01 (-0.13;0.16) |  |
| 4 | 0.01 | 2500 | 24 | -0.02 (-0.15;0.11) | -0.06 (-0.20;0.09) | -0.07 (-0.21;0.09) | -0.02 (-0.15;0.11) | -0.06 (-0.20;0.09) | -0.07 (-0.21;0.09) |  |
| 5 | 0.01 | 5000 | 3 | 0.02 (-0.08;0.12) | 0.02 (-0.08;0.12) | 0.02 (-0.08;0.12) | 0.02 (-0.08;0.12) | 0.02 (-0.08;0.12) | 0.02 (-0.08;0.12) |  |
| 6 | 0.01 | 5000 | 6 | 0.02 (-0.07;0.12) | 0.03 (-0.06;0.12) | 0.03 (-0.06;0.13) | 0.02 (-0.07;0.12) | 0.03 (-0.06;0.12) | 0.03 (-0.06;0.13) |  |
| 7 | 0.01 | 5000 | 12 | 0.01 (-0.08;0.1) | 0.01 (-0.08;0.11) | 0.01 (-0.08;0.11) | 0.01 (-0.08;0.10) | 0.01 (-0.08;0.11) | 0.01 (-0.08;0.11) |  |
| 8 | 0.01 | 5000 | 24 | -0.02 (-0.12;0.07) | -0.03 (-0.13;0.06) | -0.04 (-0.14;0.06) | -0.02 (-0.12;0.07) | -0.03 (-0.13;0.06) | -0.04 (-0.14;0.06) |  |
| 9 | 0.1 | 2500 | 3 | 0.01 (-0.04;0.05) | 0.01 (-0.04;0.05) | 0.01 (-0.04;0.05) | 0.01 (-0.04;0.05) | 0.01 (-0.04;0.05) | 0.01 (-0.04;0.05) |  |
| 10 | 0.1 | 2500 | 6 | 0.00 (-0.05;0.04) | 0.00 (-0.05;0.04) | 0.00 (-0.05;0.04) | 0.00 (-0.05;0.04) | 0.00 (-0.05;0.04) | 0.00 (-0.05;0.04) |  |
| 11 | 0.1 | 2500 | 12 | 0.00 (-0.04;0.05) | 0.00 (-0.04;0.05) | 0.00 (-0.04;0.05) | 0.00 (-0.04;0.05) | 0.00 (-0.04;0.05) | 0.00 (-0.04;0.05) |  |
| 12 | 0.1 | 2500 | 24 | -0.02 (-0.06;0.03) | -0.02 (-0.06;0.03) | -0.02 (-0.06;0.03) | -0.02 (-0.06;0.03) | -0.02 (-0.06;0.03) | -0.02 (-0.06;0.03) |  |
| 13 | 0.1 | 5000 | 3 | 0.00 (-0.03;0.03) | 0.00 (-0.03;0.03) | 0.00 (-0.03;0.03) | 0.00 (-0.03;0.03) | 0.00 (-0.03;0.03) | 0.00 (-0.03;0.03) |  |
| 14 | 0.1 | 5000 | 6 | 0.01 (-0.02;0.05) | 0.01 (-0.02;0.05) | 0.01 (-0.02;0.05) | 0.01 (-0.02;0.05) | 0.01 (-0.02;0.05) | 0.01 (-0.02;0.05) |  |
| 15 | 0.1 | 5000 | 12 | 0.00 (-0.04;0.03) | 0.00 (-0.04;0.03) | 0.00 (-0.04;0.03) | 0.00 (-0.04;0.03) | 0.00 (-0.04;0.03) | 0.00 (-0.04;0.03) |  |
| 16 | 0.1 | 5000 | 24 | 0.00 (-0.03;0.03) | 0.00 (-0.03;0.03) | 0.00 (-0.03;0.03) | 0.00 (-0.03;0.03) | 0.00 (-0.03;0.03) | 0.00 (-0.03;0.03) |  |
| 17 | 0.3 | 2500 | 3 | 0.01 (-0.03;0.04) | 0.01 (-0.03;0.04) | 0.01 (-0.03;0.04) | 0.01 (-0.03;0.04) | 0.01 (-0.03;0.04) | 0.01 (-0.03;0.04) |  |
| 18 | 0.3 | 2500 | 6 | 0.00 (-0.03;0.03) | 0.00 (-0.03;0.03) | 0.00 (-0.03;0.03) | 0.00 (-0.03;0.03) | 0.00 (-0.03;0.03) | 0.00 (-0.03;0.03) |  |
| 19 | 0.3 | 2500 | 12 | 0.00 (-0.04;0.03) | 0.00 (-0.04;0.03) | 0.00 (-0.04;0.03) | 0.00 (-0.04;0.03) | 0.00 (-0.04;0.03) | 0.00 (-0.04;0.03) |  |
| 20 | 0.3 | 2500 | 24 | 0.01 (-0.03;0.04) | 0.01 (-0.03;0.04) | 0.01 (-0.03;0.04) | 0.01 (-0.03;0.04) | 0.01 (-0.03;0.04) | 0.01 (-0.03;0.04) |  |
| 21 | 0.3 | 5000 | 3 | 0.00 (-0.02;0.02) | 0.00 (-0.02;0.02) | 0.00 (-0.02;0.02) | 0.00 (-0.02;0.02) | 0.00 (-0.02;0.02) | 0.00 (-0.02;0.02) |  |
| 22 | 0.3 | 5000 | 6 | 0.00 (-0.02;0.03) | 0.00 (-0.02;0.03) | 0.00 (-0.02;0.03) | 0.00 (-0.02;0.03) | 0.00 (-0.02;0.03) | 0.00 (-0.02;0.03) |  |
| 23 | 0.3 | 5000 | 12 | 0.00 (-0.02;0.02) | 0.00 (-0.02;0.02) | 0.00 (-0.02;0.02) | 0.00 (-0.02;0.02) | 0.00 (-0.02;0.02) | 0.00 (-0.02;0.02) |  |
| 24 | 0.3 | 5000 | 24 | -0.01 (-0.03;0.01) | -0.01 (-0.03;0.01) | -0.01 (-0.03;0.01) | -0.01 (-0.03;0.01) | -0.01 (-0.03;0.01) | -0.01 (-0.03;0.01) |  |

Table S6. Test set calibration slope, reported as median (IQR) over the 2000 runs for SLR and Ridge in the 24 simulation scenarios.

|  |  |  |  | **SLR** | | | | **RIDGE** | | | |
| --- | --- | --- | --- | --- | --- | --- | --- | --- | --- | --- | --- |
| **Scenario** | **EF** | **N** | **p** | **Uncorrected** | **RUS** | **ROS** | **SMOTE** | **Uncorrected** | **RUS** | **ROS** | **SMOTE** |
| 1 | 0.01 | 2500 | 3 | 0.89 (0.77;1.05) | 0.71 (0.54;0.90) | 0.83 (0.70;1.01) | 0.71 (0.60;0.87) | 1.09 (0.90;1.39) | 1.23 (0.88;2.00) | 0.84 (0.71;1.02) | 0.72 (0.60;0.88) |
| 2 | 0.01 | 2500 | 6 | 0.85 (0.74;0.96) | 0.54 (0.42;0.69) | 0.75 (0.64;0.89) | 0.63 (0.54;0.74) | 1.11 (0.93;1.37) | 1.22 (0.86;1.86) | 0.76 (0.64;0.90) | 0.64 (0.55;0.75) |
| 3 | 0.01 | 2500 | 12 | 0.74 (0.66;0.83) | 0.32 (0.22;0.43) | 0.60 (0.50;0.70) | 0.50 (0.43;0.59) | 1.11 (0.94;1.38) | 1.21 (0.85;1.90) | 0.61 (0.51;0.72) | 0.51 (0.44;0.60) |
| 4 | 0.01 | 2500 | 24 | 0.48 (0.41;0.54) | 0.01 (0.01;0.06) | 0.31 (0.26;0.38) | 0.28 (0.23;0.33) | 1.07 (0.85;1.48) | 1.13 (0.69;2.36) | 0.32 (0.26;0.39) | 0.29 (0.24;0.34) |
| 5 | 0.01 | 5000 | 3 | 0.95 (0.85;1.06) | 0.83 (0.69;1.00) | 0.91 (0.81;1.04) | 0.81 (0.71;0.93) | 1.06 (0.94;1.20) | 1.12 (0.91;1.47) | 0.91 (0.81;1.04) | 0.81 (0.71;0.93) |
| 6 | 0.01 | 5000 | 6 | 0.93 (0.85;1.02) | 0.75 (0.63;0.89) | 0.88 (0.78;0.99) | 0.76 (0.67;0.85) | 1.07 (0.97;1.20) | 1.16 (0.93;1.47) | 0.88 (0.79;0.99) | 0.76 (0.68;0.86) |
| 7 | 0.01 | 5000 | 12 | 0.86 (0.79;0.94) | 0.57 (0.48;0.67) | 0.77 (0.70;0.86) | 0.65 (0.59;0.73) | 1.07 (0.97;1.23) | 1.15 (0.92;1.49) | 0.78 (0.70;0.87) | 0.66 (0.59;0.73) |
| 8 | 0.01 | 5000 | 24 | 0.65 (0.60;0.71) | 0.26 (0.20;0.33) | 0.53 (0.46;0.59) | 0.44 (0.39;0.49) | 1.04 (0.91;1.22) | 1.10 (0.83;1.57) | 0.53 (0.47;0.60) | 0.45 (0.39;0.50) |
| 9 | 0.1 | 2500 | 3 | 1.00 (0.94;1.05) | 0.98 (0.91;1.06) | 0.99 (0.93;1.06) | 0.95 (0.89;1.00) | 1.03 (0.97;1.09) | 1.05 (0.96;1.14) | 1.00 (0.94;1.07) | 0.95 (0.89;1.01) |
| 10 | 0.1 | 2500 | 6 | 0.96 (0.92;1.02) | 0.93 (0.86;1.00) | 0.95 (0.90;1.01) | 0.87 (0.82;0.93) | 1.01 (0.96;1.07) | 1.03 (0.95;1.12) | 0.97 (0.91;1.03) | 0.88 (0.83;0.94) |
| 11 | 0.1 | 2500 | 12 | 0.93 (0.89;0.98) | 0.87 (0.81;0.93) | 0.92 (0.87;0.97) | 0.81 (0.77;0.86) | 1.01 (0.96;1.06) | 1.04 (0.95;1.12) | 0.94 (0.88;0.99) | 0.83 (0.78;0.88) |
| 12 | 0.1 | 2500 | 24 | 0.85 (0.81;0.89) | 0.73 (0.68;0.79) | 0.82 (0.77;0.86) | 0.71 (0.67;0.75) | 0.99 (0.94;1.06) | 1.03 (0.93;1.13) | 0.85 (0.80;0.90) | 0.74 (0.70;0.78) |
| 13 | 0.1 | 5000 | 3 | 0.99 (0.96;1.03) | 0.98 (0.93;1.04) | 0.99 (0.95;1.04) | 0.95 (0.92;1.00) | 1.01 (0.97;1.05) | 1.02 (0.96;1.08) | 1.00 (0.95;1.04) | 0.96 (0.92;1.00) |
| 14 | 0.1 | 5000 | 6 | 0.99 (0.96;1.03) | 0.97 (0.92;1.02) | 0.99 (0.95;1.03) | 0.91 (0.87;0.95) | 1.02 (0.98;1.05) | 1.02 (0.96;1.08) | 0.99 (0.95;1.04) | 0.92 (0.88;0.96) |
| 15 | 0.1 | 5000 | 12 | 0.97 (0.94;1.00) | 0.93 (0.89;0.98) | 0.96 (0.92;1.00) | 0.86 (0.82;0.89) | 1.01 (0.97;1.05) | 1.02 (0.97;1.08) | 0.97 (0.93;1.01) | 0.87 (0.83;0.90) |
| 16 | 0.1 | 5000 | 24 | 0.93 (0.90;0.96) | 0.86 (0.82;0.90) | 0.91 (0.88;0.94) | 0.79 (0.76;0.82) | 1.00 (0.97;1.04) | 1.03 (0.97;1.09) | 0.93 (0.89;0.96) | 0.81 (0.78;0.84) |
| 17 | 0.3 | 2500 | 3 | 0.99 (0.96;1.03) | 0.99 (0.95;1.04) | 0.99 (0.95;1.04) | 0.98 (0.94;1.01) | 1.01 (0.97;1.05) | 1.01 (0.97;1.06) | 1.00 (0.96;1.05) | 0.99 (0.95;1.02) |
| 18 | 0.3 | 2500 | 6 | 0.99 (0.96;1.03) | 0.99 (0.94;1.03) | 0.99 (0.95;1.03) | 0.95 (0.92;0.99) | 1.02 (0.98;1.06) | 1.02 (0.97;1.07) | 1.00 (0.97;1.05) | 0.97 (0.93;1.01) |
| 19 | 0.3 | 2500 | 12 | 0.97 (0.93;1.00) | 0.95 (0.91;0.99) | 0.96 (0.92;0.99) | 0.90 (0.87;0.94) | 1.01 (0.97;1.04) | 1.01 (0.97;1.06) | 0.98 (0.95;1.02) | 0.93 (0.90;0.97) |
| 20 | 0.3 | 2500 | 24 | 0.94 (0.91;0.98) | 0.91 (0.88;0.96) | 0.93 (0.89;0.97) | 0.87 (0.83;0.90) | 1.03 (0.99;1.07) | 1.04 (0.98;1.09) | 0.98 (0.94;1.02) | 0.92 (0.88;0.96) |
| 21 | 0.3 | 5000 | 3 | 1.00 (0.98;1.03) | 1.00 (0.97;1.03) | 1.00 (0.97;1.03) | 0.99 (0.96;1.02) | 1.01 (0.98;1.04) | 1.01 (0.98;1.04) | 1.01 (0.98;1.04) | 1.00 (0.97;1.02) |
| 22 | 0.3 | 5000 | 6 | 0.98 (0.95;1.01) | 0.98 (0.94;1.01) | 0.98 (0.95;1.01) | 0.94 (0.92;0.97) | 0.99 (0.96;1.02) | 0.99 (0.96;1.02) | 0.99 (0.96;1.02) | 0.95 (0.93;0.98) |
| 23 | 0.3 | 5000 | 12 | 0.99 (0.96;1.01) | 0.98 (0.95;1.01) | 0.98 (0.96;1.01) | 0.93 (0.90;0.95) | 1.01 (0.98;1.03) | 1.01 (0.98;1.04) | 1.00 (0.97;1.02) | 0.94 (0.92;0.97) |
| 24 | 0.3 | 5000 | 24 | 0.97 (0.95;1.00) | 0.96 (0.93;0.99) | 0.97 (0.94;0.99) | 0.90 (0.88;0.93) | 1.02 (0.99;1.05) | 1.02 (0.98;1.06) | 0.99 (0.96;1.02) | 0.93 (0.90;0.96) |

Table S7. Test set sensitivity reported as median (IQR) over the 2000 runs for SLR and Ridge in the 24 simulation scenarios. For uncorrected training sets, we used either the default threshold of 0.5 or a threshold based on the true event fraction. For RUS/ROS/SMOTE, the default threshold of 0.5 was used.

|  |  |  |  | **Imbalance correction method (threshold)** | | | | |
| --- | --- | --- | --- | --- | --- | --- | --- | --- |
| **Scenario** | **EF** | **N** | **p** | **Uncorrected (0.5)** | **Uncorrected (EF)** | **RUS (0.5)** | **ROS (0.5)** | **SMOTE (0.5)** |
| SLR | | | | | | | | |
| 1 | 0.01 | 2500 | 3 | 0 (0;0) | 0.60 (0.53;0.67) | 0.64 (0.60;0.68) | 0.63 (0.59;0.66) | 0.62 (0.59;0.65) |
| 2 | 0.01 | 2500 | 6 | 0 (0;0) | 0.66 (0.60;0.73) | 0.66 (0.62;0.71) | 0.65 (0.61;0.67) | 0.63 (0.60;0.66) |
| 3 | 0.01 | 2500 | 12 | 0 (0;0) | 0.67 (0.61;0.72) | 0.65 (0.61;0.69) | 0.62 (0.58;0.65) | 0.60 (0.56;0.64) |
| 4 | 0.01 | 2500 | 24 | 0 (0;0) | 0.52 (0.46;0.59) | 0.58 (0.53;0.63) | 0.48 (0.44;0.52) | 0.46 (0.41;0.50) |
| 5 | 0.01 | 5000 | 3 | 0 (0;0) | 0.62 (0.57;0.67) | 0.65 (0.62;0.68) | 0.65 (0.62;0.67) | 0.64 (0.62;0.66) |
| 6 | 0.01 | 5000 | 6 | 0 (0;0) | 0.69 (0.65;0.73) | 0.68 (0.65;0.71) | 0.67 (0.65;0.69) | 0.66 (0.64;0.68) |
| 7 | 0.01 | 5000 | 12 | 0 (0;0) | 0.69 (0.65;0.72) | 0.67 (0.64;0.69) | 0.65 (0.63;0.67) | 0.64 (0.61;0.66) |
| 8 | 0.01 | 5000 | 24 | 0 (0;0) | 0.60 (0.56;0.65) | 0.62 (0.59;0.65) | 0.57 (0.55;0.60) | 0.55 (0.52;0.58) |
| 9 | 0.1 | 2500 | 3 | 0.02 (0.01;0.02) | 0.67 (0.65;0.69) | 0.67 (0.66;0.68) | 0.67 (0.66;0.67) | 0.64 (0.63;0.66) |
| 10 | 0.1 | 2500 | 6 | 0.03 (0.02;0.04) | 0.70 (0.68;0.71) | 0.68 (0.67;0.69) | 0.68 (0.67;0.68) | 0.65 (0.64;0.67) |
| 11 | 0.1 | 2500 | 12 | 0.05 (0.04;0.06) | 0.71 (0.69;0.72) | 0.68 (0.67;0.69) | 0.68 (0.67;0.69) | 0.66 (0.64;0.67) |
| 12 | 0.1 | 2500 | 24 | 0.04 (0.03;0.05) | 0.67 (0.65;0.69) | 0.66 (0.65;0.67) | 0.65 (0.64;0.66) | 0.62 (0.61;0.63) |
| 13 | 0.1 | 5000 | 3 | 0.02 (0.01;0.02) | 0.67 (0.65;0.68) | 0.67 (0.66;0.68) | 0.67 (0.66;0.67) | 0.64 (0.63;0.66) |
| 14 | 0.1 | 5000 | 6 | 0.03 (0.03;0.04) | 0.70 (0.69;0.72) | 0.68 (0.68;0.69) | 0.68 (0.68;0.69) | 0.66 (0.65;0.67) |
| 15 | 0.1 | 5000 | 12 | 0.05 (0.04;0.05) | 0.71 (0.70;0.73) | 0.69 (0.68;0.69) | 0.69 (0.68;0.69) | 0.66 (0.65;0.67) |
| 16 | 0.1 | 5000 | 24 | 0.03 (0.03;0.04) | 0.68 (0.67;0.70) | 0.67 (0.66;0.68) | 0.67 (0.66;0.67) | 0.64 (0.63;0.65) |
| 17 | 0.3 | 2500 | 3 | 0.31 (0.30;0.33) | 0.68 (0.66;0.69) | 0.67 (0.67;0.68) | 0.67 (0.67;0.68) | 0.61 (0.60;0.63) |
| 18 | 0.3 | 2500 | 6 | 0.32 (0.31;0.34) | 0.68 (0.67;0.69) | 0.67 (0.67;0.68) | 0.67 (0.67;0.68) | 0.62 (0.61;0.63) |
| 19 | 0.3 | 2500 | 12 | 0.35 (0.33;0.36) | 0.69 (0.68;0.70) | 0.68 (0.67;0.69) | 0.68 (0.67;0.69) | 0.63 (0.62;0.64) |
| 20 | 0.3 | 2500 | 24 | 0.33 (0.31;0.34) | 0.68 (0.66;0.69) | 0.67 (0.67;0.68) | 0.67 (0.66;0.67) | 0.61 (0.60;0.63) |
| 21 | 0.3 | 5000 | 3 | 0.31 (0.30;0.32) | 0.68 (0.67;0.69) | 0.68 (0.67;0.68) | 0.68 (0.67;0.68) | 0.61 (0.60;0.62) |
| 22 | 0.3 | 5000 | 6 | 0.32 (0.31;0.33) | 0.68 (0.67;0.69) | 0.67 (0.67;0.68) | 0.67 (0.67;0.68) | 0.61 (0.61;0.62) |
| 23 | 0.3 | 5000 | 12 | 0.35 (0.34;0.36) | 0.69 (0.68;0.70) | 0.68 (0.68;0.69) | 0.68 (0.68;0.69) | 0.63 (0.63;0.64) |
| 24 | 0.3 | 5000 | 24 | 0.33 (0.31;0.34) | 0.68 (0.67;0.69) | 0.68 (0.67;0.68) | 0.68 (0.67;0.68) | 0.62 (0.61;0.63) |
| RIDGE | | | | | | | | |
| 1 | 0.01 | 2500 | 3 | 0 (0;0) | 0.62 (0.54;0.70) | 0.64 (0.60;0.68) | 0.63 (0.59;0.66) | 0.62 (0.59;0.65) |
| 2 | 0.01 | 2500 | 6 | 0 (0;0) | 0.71 (0.64;0.78) | 0.67 (0.63;0.71) | 0.65 (0.61;0.67) | 0.63 (0.60;0.66) |
| 3 | 0.01 | 2500 | 12 | 0 (0;0) | 0.75 (0.68;0.81) | 0.67 (0.63;0.71) | 0.62 (0.58;0.65) | 0.60 (0.56;0.64) |
| 4 | 0.01 | 2500 | 24 | 0 (0;0) | 0.67 (0.57;0.76) | 0.61 (0.57;0.65) | 0.48 (0.44;0.52) | 0.46 (0.41;0.50) |
| 5 | 0.01 | 5000 | 3 | 0 (0;0) | 0.63 (0.58;0.69) | 0.65 (0.62;0.68) | 0.65 (0.62;0.67) | 0.64 (0.62;0.66) |
| 6 | 0.01 | 5000 | 6 | 0 (0;0) | 0.72 (0.68;0.75) | 0.68 (0.65;0.71) | 0.67 (0.65;0.69) | 0.66 (0.64;0.68) |
| 7 | 0.01 | 5000 | 12 | 0 (0;0) | 0.73 (0.69;0.77) | 0.67 (0.65;0.70) | 0.65 (0.63;0.67) | 0.64 (0.61;0.66) |
| 8 | 0.01 | 5000 | 24 | 0 (0;0) | 0.68 (0.63;0.74) | 0.63 (0.60;0.66) | 0.57 (0.55;0.60) | 0.55 (0.52;0.58) |
| 9 | 0.1 | 2500 | 3 | 0.01 (0.01;0.02) | 0.67 (0.65;0.69) | 0.67 (0.66;0.68) | 0.67 (0.66;0.67) | 0.64 (0.63;0.66) |
| 10 | 0.1 | 2500 | 6 | 0.03 (0.02;0.03) | 0.70 (0.68;0.72) | 0.68 (0.67;0.69) | 0.68 (0.67;0.68) | 0.65 (0.64;0.67) |
| 11 | 0.1 | 2500 | 12 | 0.03 (0.03;0.04) | 0.72 (0.70;0.74) | 0.68 (0.67;0.69) | 0.68 (0.67;0.69) | 0.65 (0.64;0.67) |
| 12 | 0.1 | 2500 | 24 | 0.02 (0.01;0.02) | 0.69 (0.67;0.71) | 0.66 (0.65;0.67) | 0.65 (0.64;0.66) | 0.62 (0.61;0.63) |
| 13 | 0.1 | 5000 | 3 | 0.02 (0.01;0.02) | 0.67 (0.66;0.68) | 0.67 (0.66;0.68) | 0.67 (0.66;0.67) | 0.64 (0.63;0.66) |
| 14 | 0.1 | 5000 | 6 | 0.03 (0.02;0.04) | 0.70 (0.69;0.72) | 0.68 (0.68;0.69) | 0.68 (0.68;0.69) | 0.66 (0.65;0.67) |
| 15 | 0.1 | 5000 | 12 | 0.04 (0.03;0.05) | 0.72 (0.71;0.73) | 0.69 (0.68;0.69) | 0.69 (0.68;0.69) | 0.66 (0.65;0.67) |
| 16 | 0.1 | 5000 | 24 | 0.02 (0.02;0.03) | 0.69 (0.68;0.71) | 0.67 (0.66;0.68) | 0.67 (0.66;0.67) | 0.64 (0.63;0.65) |
| 17 | 0.3 | 2500 | 3 | 0.31 (0.29;0.33) | 0.68 (0.66;0.69) | 0.67 (0.67;0.68) | 0.67 (0.67;0.68) | 0.61 (0.60;0.63) |
| 18 | 0.3 | 2500 | 6 | 0.32 (0.30;0.33) | 0.68 (0.67;0.69) | 0.67 (0.67;0.68) | 0.67 (0.67;0.68) | 0.62 (0.60;0.63) |
| 19 | 0.3 | 2500 | 12 | 0.34 (0.32;0.35) | 0.69 (0.68;0.71) | 0.68 (0.67;0.69) | 0.68 (0.67;0.69) | 0.63 (0.62;0.64) |
| 20 | 0.3 | 2500 | 24 | 0.30 (0.28;0.32) | 0.68 (0.67;0.69) | 0.67 (0.67;0.68) | 0.67 (0.66;0.67) | 0.61 (0.60;0.63) |
| 21 | 0.3 | 5000 | 3 | 0.31 (0.30;0.32) | 0.68 (0.67;0.69) | 0.68 (0.67;0.68) | 0.68 (0.67;0.68) | 0.61 (0.60;0.62) |
| 22 | 0.3 | 5000 | 6 | 0.31 (0.30;0.32) | 0.68 (0.67;0.69) | 0.67 (0.67;0.68) | 0.67 (0.67;0.68) | 0.61 (0.61;0.62) |
| 23 | 0.3 | 5000 | 12 | 0.34 (0.33;0.35) | 0.69 (0.69;0.70) | 0.68 (0.68;0.69) | 0.68 (0.68;0.69) | 0.63 (0.63;0.64) |
| 24 | 0.3 | 5000 | 24 | 0.31 (0.30;0.33) | 0.68 (0.68;0.69) | 0.68 (0.67;0.68) | 0.68 (0.67;0.68) | 0.62 (0.61;0.63) |

Table S8. Test set specificity reported as median (IQR) over the 2000 runs for SLR and Ridge in the 24 simulation scenarios. For uncorrected training sets, we used either the default threshold of 0.5 or a threshold based on the true event fraction. For RUS/ROS/SMOTE, the default threshold of 0.5 was used.

|  |  |  |  | **Imbalance correction method (threshold)** | | | | |
| --- | --- | --- | --- | --- | --- | --- | --- | --- |
| **Scenario** | **EF** | **N** | **p** | **Uncorrected (0.5)** | **Uncorrected (EF)** | **RUS (0.5)** | **ROS (0.5)** | **SMOTE (0.5)** |
| SLR | | | | | | | | |
| 1 | 0.01 | 2500 | 3 | 1 (1;1) | 0.70 (0.64;0.76) | 0.65 (0.60;0.68) | 0.67 (0.65;0.70) | 0.68 (0.65;0.71) |
| 2 | 0.01 | 2500 | 6 | 1 (1;1) | 0.69 (0.63;0.74) | 0.65 (0.60;0.69) | 0.70 (0.68;0.72) | 0.71 (0.68;0.74) |
| 3 | 0.01 | 2500 | 12 | 1 (1;1) | 0.70 (0.65;0.74) | 0.63 (0.59;0.67) | 0.73 (0.70;0.75) | 0.74 (0.72;0.76) |
| 4 | 0.01 | 2500 | 24 | 1 (1;1) | 0.74 (0.69;0.78) | 0.57 (0.52;0.62) | 0.75 (0.73;0.77) | 0.77 (0.75;0.80) |
| 5 | 0.01 | 5000 | 3 | 1 (1;1) | 0.70 (0.65;0.74) | 0.66 (0.63;0.68) | 0.67 (0.66;0.69) | 0.68 (0.66;0.69) |
| 6 | 0.01 | 5000 | 6 | 1 (1;1) | 0.68 (0.64;0.72) | 0.67 (0.64;0.69) | 0.69 (0.68;0.71) | 0.70 (0.68;0.72) |
| 7 | 0.01 | 5000 | 12 | 1 (1;1) | 0.68 (0.64;0.72) | 0.66 (0.63;0.68) | 0.71 (0.69;0.73) | 0.72 (0.70;0.74) |
| 8 | 0.01 | 5000 | 24 | 1 (1;1) | 0.71 (0.67;0.74) | 0.62 (0.59;0.65) | 0.72 (0.70;0.74) | 0.74 (0.72;0.75) |
| 9 | 0.1 | 2500 | 3 | 1 (1;1) | 0.67 (0.64;0.68) | 0.66 (0.65;0.67) | 0.66 (0.66;0.67) | 0.69 (0.68;0.70) |
| 10 | 0.1 | 2500 | 6 | 1 (1;1) | 0.66 (0.64;0.68) | 0.67 (0.66;0.69) | 0.68 (0.67;0.69) | 0.70 (0.69;0.71) |
| 11 | 0.1 | 2500 | 12 | 1 (0.99;1) | 0.66 (0.64;0.68) | 0.68 (0.67;0.69) | 0.69 (0.68;0.70) | 0.71 (0.70;0.72) |
| 12 | 0.1 | 2500 | 24 | 1 (0.99;1) | 0.66 (0.65;0.68) | 0.66 (0.65;0.67) | 0.68 (0.67;0.69) | 0.70 (0.69;0.71) |
| 13 | 0.1 | 5000 | 3 | 1 (1;1) | 0.66 (0.65;0.68) | 0.66 (0.65;0.67) | 0.66 (0.66;0.67) | 0.69 (0.68;0.70) |
| 14 | 0.1 | 5000 | 6 | 1 (1;1) | 0.66 (0.65;0.68) | 0.68 (0.67;0.68) | 0.68 (0.67;0.68) | 0.70 (0.69;0.71) |
| 15 | 0.1 | 5000 | 12 | 1 (0.99;1) | 0.66 (0.64;0.67) | 0.68 (0.67;0.69) | 0.69 (0.68;0.69) | 0.71 (0.70;0.72) |
| 16 | 0.1 | 5000 | 24 | 1 (1;1) | 0.66 (0.65;0.68) | 0.67 (0.66;0.68) | 0.68 (0.67;0.69) | 0.70 (0.69;0.71) |
| 17 | 0.3 | 2500 | 3 | 0.92 (0.91;0.92) | 0.67 (0.66;0.68) | 0.67 (0.67;0.68) | 0.67 (0.67;0.68) | 0.73 (0.72;0.74) |
| 18 | 0.3 | 2500 | 6 | 0.92 (0.91;0.92) | 0.67 (0.66;0.68) | 0.67 (0.67;0.68) | 0.67 (0.67;0.68) | 0.73 (0.72;0.74) |
| 19 | 0.3 | 2500 | 12 | 0.91 (0.90;0.91) | 0.67 (0.66;0.68) | 0.67 (0.67;0.68) | 0.68 (0.67;0.68) | 0.72 (0.71;0.73) |
| 20 | 0.3 | 2500 | 24 | 0.91 (0.90;0.92) | 0.67 (0.65;0.68) | 0.66 (0.66;0.67) | 0.67 (0.66;0.68) | 0.72 (0.71;0.73) |
| 21 | 0.3 | 5000 | 3 | 0.92 (0.91;0.92) | 0.67 (0.66;0.68) | 0.67 (0.67;0.68) | 0.67 (0.67;0.68) | 0.73 (0.72;0.74) |
| 22 | 0.3 | 5000 | 6 | 0.91 (0.91;0.92) | 0.67 (0.66;0.67) | 0.67 (0.67;0.67) | 0.67 (0.67;0.67) | 0.72 (0.72;0.73) |
| 23 | 0.3 | 5000 | 12 | 0.91 (0.90;0.91) | 0.67 (0.66;0.68) | 0.68 (0.67;0.68) | 0.68 (0.67;0.68) | 0.72 (0.72;0.73) |
| 24 | 0.3 | 5000 | 24 | 0.91 (0.91;0.92) | 0.67 (0.66;0.68) | 0.67 (0.66;0.67) | 0.67 (0.67;0.68) | 0.72 (0.72;0.73) |
| RIDGE | | | | | | | | |
| 1 | 0.01 | 2500 | 3 | 1 (1;1) | 0.68 (0.61;0.75) | 0.65 (0.60;0.68) | 0.67 (0.65;0.70) | 0.68 (0.65;0.71) |
| 2 | 0.01 | 2500 | 6 | 1 (1;1) | 0.65 (0.57;0.71) | 0.65 (0.61;0.69) | 0.70 (0.68;0.72) | 0.71 (0.68;0.74) |
| 3 | 0.01 | 2500 | 12 | 1 (1;1) | 0.62 (0.54;0.69) | 0.64 (0.60;0.68) | 0.73 (0.70;0.75) | 0.74 (0.72;0.76) |
| 4 | 0.01 | 2500 | 24 | 1 (1;1) | 0.61 (0.52;0.69) | 0.61 (0.57;0.64) | 0.75 (0.73;0.77) | 0.77 (0.75;0.80) |
| 5 | 0.01 | 5000 | 3 | 1 (1;1) | 0.68 (0.63;0.73) | 0.66 (0.63;0.68) | 0.67 (0.66;0.69) | 0.68 (0.66;0.69) |
| 6 | 0.01 | 5000 | 6 | 1 (1;1) | 0.65 (0.61;0.69) | 0.67 (0.64;0.69) | 0.69 (0.68;0.71) | 0.70 (0.68;0.72) |
| 7 | 0.01 | 5000 | 12 | 1 (1;1) | 0.63 (0.59;0.68) | 0.66 (0.64;0.69) | 0.71 (0.69;0.73) | 0.72 (0.70;0.74) |
| 8 | 0.01 | 5000 | 24 | 1 (1;1) | 0.63 (0.57;0.68) | 0.63 (0.60;0.66) | 0.72 (0.70;0.74) | 0.74 (0.72;0.75) |
| 9 | 0.1 | 2500 | 3 | 1 (1;1) | 0.66 (0.64;0.68) | 0.66 (0.65;0.67) | 0.66 (0.66;0.67) | 0.69 (0.68;0.70) |
| 10 | 0.1 | 2500 | 6 | 1 (1;1) | 0.66 (0.64;0.68) | 0.67 (0.66;0.69) | 0.68 (0.67;0.69) | 0.70 (0.69;0.71) |
| 11 | 0.1 | 2500 | 12 | 1 (1;1) | 0.65 (0.63;0.67) | 0.68 (0.67;0.69) | 0.69 (0.68;0.70) | 0.71 (0.70;0.72) |
| 12 | 0.1 | 2500 | 24 | 1 (1;1) | 0.64 (0.62;0.66) | 0.66 (0.65;0.67) | 0.68 (0.67;0.69) | 0.70 (0.69;0.72) |
| 13 | 0.1 | 5000 | 3 | 1 (1;1) | 0.66 (0.65;0.68) | 0.66 (0.65;0.67) | 0.66 (0.66;0.67) | 0.69 (0.68;0.70) |
| 14 | 0.1 | 5000 | 6 | 1 (1;1) | 0.66 (0.64;0.67) | 0.68 (0.67;0.68) | 0.68 (0.67;0.68) | 0.70 (0.69;0.71) |
| 15 | 0.1 | 5000 | 12 | 1 (1;1) | 0.65 (0.64;0.66) | 0.68 (0.67;0.69) | 0.69 (0.68;0.69) | 0.71 (0.70;0.72) |
| 16 | 0.1 | 5000 | 24 | 1 (1;1) | 0.65 (0.64;0.67) | 0.67 (0.66;0.68) | 0.68 (0.67;0.69) | 0.70 (0.69;0.71) |
| 17 | 0.3 | 2500 | 3 | 0.92 (0.91;0.93) | 0.67 (0.66;0.68) | 0.67 (0.67;0.68) | 0.67 (0.67;0.68) | 0.73 (0.72;0.74) |
| 18 | 0.3 | 2500 | 6 | 0.92 (0.91;0.93) | 0.67 (0.66;0.68) | 0.67 (0.67;0.68) | 0.67 (0.67;0.68) | 0.73 (0.72;0.74) |
| 19 | 0.3 | 2500 | 12 | 0.91 (0.90;0.92) | 0.67 (0.65;0.68) | 0.67 (0.67;0.68) | 0.68 (0.67;0.68) | 0.72 (0.71;0.74) |
| 20 | 0.3 | 2500 | 24 | 0.92 (0.91;0.93) | 0.66 (0.65;0.68) | 0.66 (0.66;0.67) | 0.67 (0.66;0.68) | 0.72 (0.71;0.74) |
| 21 | 0.3 | 5000 | 3 | 0.92 (0.91;0.92) | 0.67 (0.66;0.68) | 0.67 (0.67;0.68) | 0.67 (0.67;0.68) | 0.73 (0.72;0.74) |
| 22 | 0.3 | 5000 | 6 | 0.92 (0.91;0.92) | 0.67 (0.66;0.67) | 0.67 (0.67;0.67) | 0.67 (0.67;0.67) | 0.72 (0.72;0.73) |
| 23 | 0.3 | 5000 | 12 | 0.91 (0.91;0.92) | 0.67 (0.66;0.68) | 0.68 (0.67;0.68) | 0.68 (0.67;0.68) | 0.72 (0.72;0.73) |
| 24 | 0.3 | 5000 | 24 | 0.92 (0.91;0.92) | 0.66 (0.65;0.67) | 0.67 (0.66;0.67) | 0.67 (0.67;0.68) | 0.72 (0.72;0.73) |

Figure S1. Test set AUROC for the Ridge models in the simulation scenarios with an event fraction of 10%.


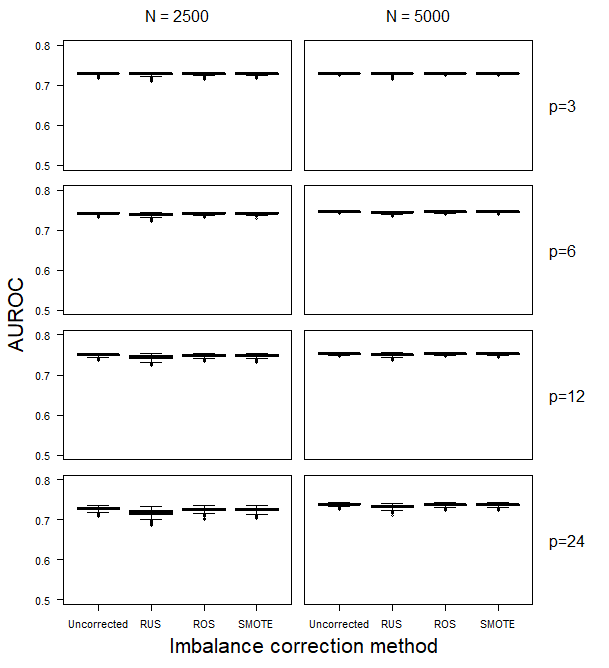


Figure S2. Test set AUROC for the Ridge models in the simulation scenarios with an event fraction of 30%.


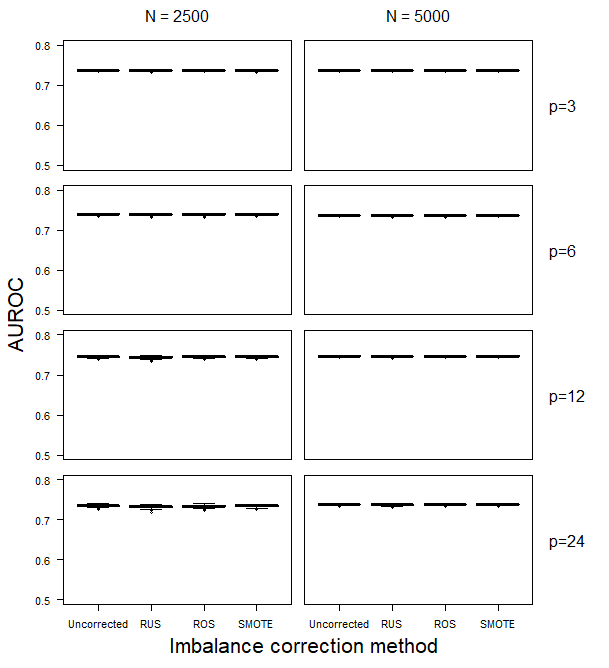


Figure S3. Test set AUROC for the SLR models in the simulation scenarios with an event fraction of 1%.


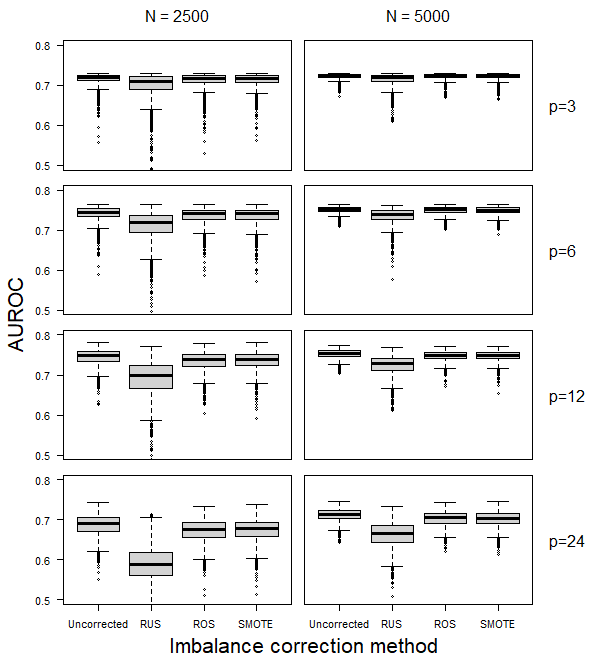


Figure S4. Test set AUROC for the SLR models in the simulation scenarios with an event fraction of 10%.


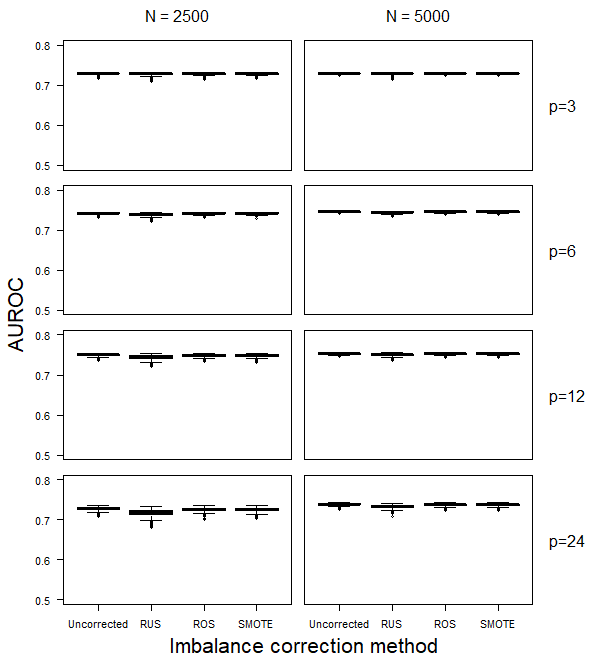


Figure S5. Test set AUROC for the SLR models in the simulation scenarios with an event fraction of 30%.


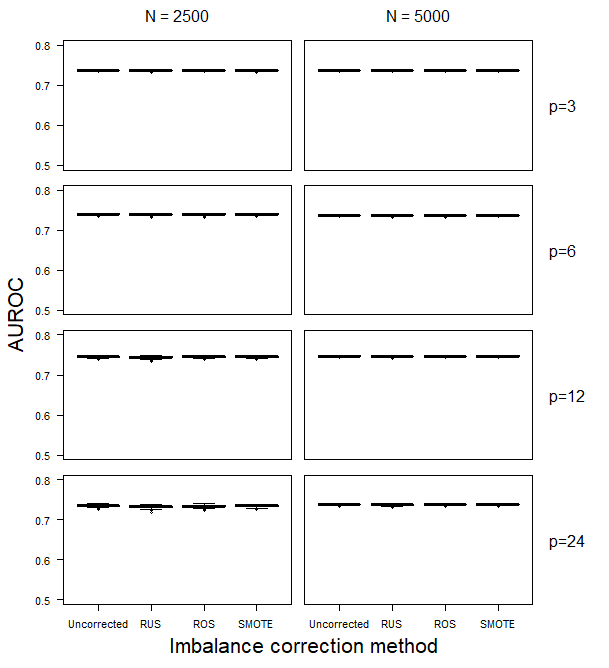


Figure S6. Test set calibration intercept for the Ridge models in the simulation scenarios with an event fraction of 10%.


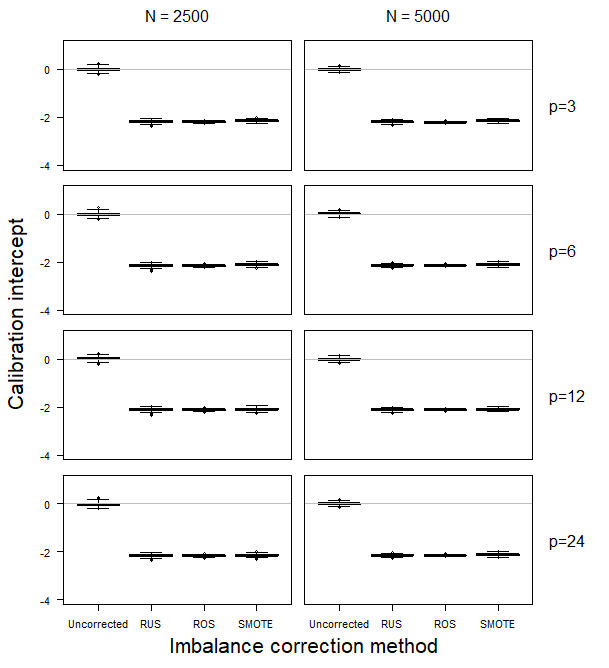


Figure S7. Test set calibration intercept for the Ridge models in the simulation scenarios with an event fraction of 30%.


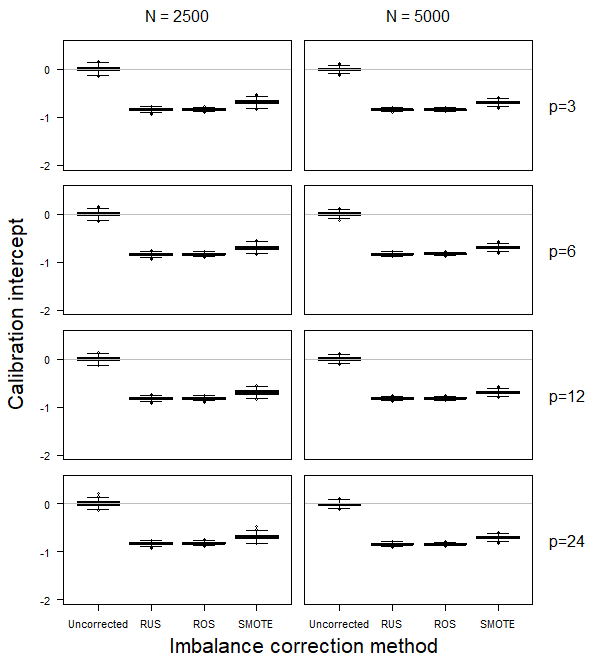


Figure S8. Test set calibration intercept for the SLR models in the simulation scenarios with an event fraction of 1%.


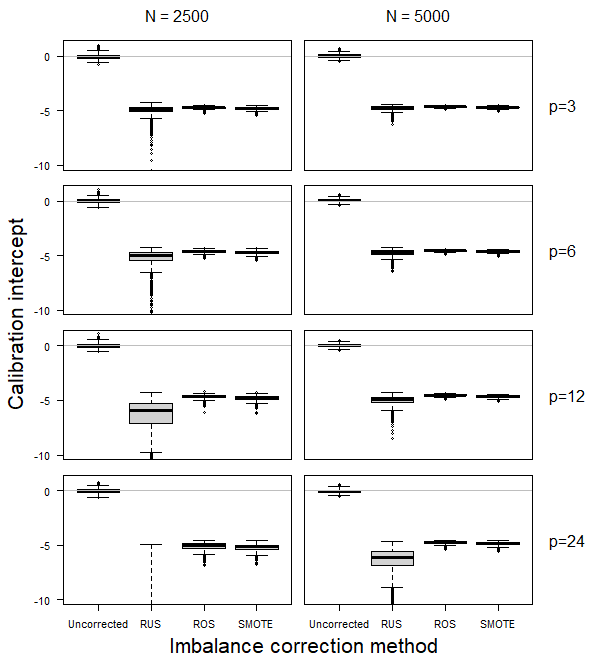


Figure S9. Test set calibration intercept for the SLR models in the simulation scenarios with an event fraction of 10%.


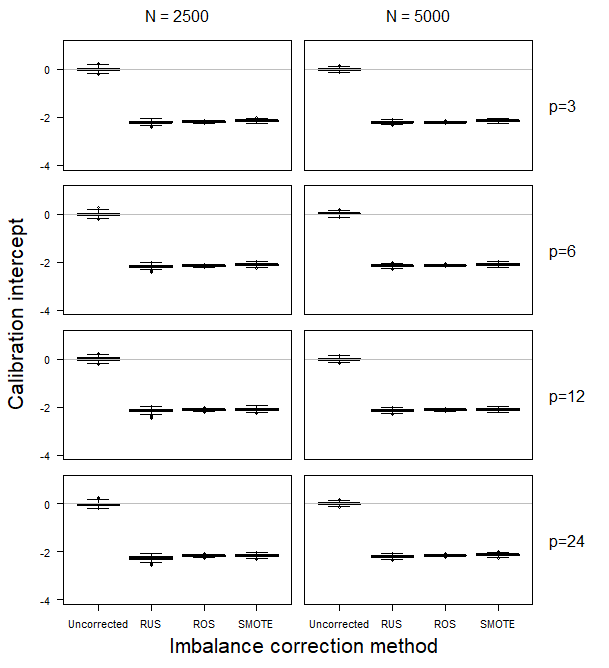


Figure S10. Test set calibration intercept for the SLR models in the simulation scenarios with an event fraction of 30%.


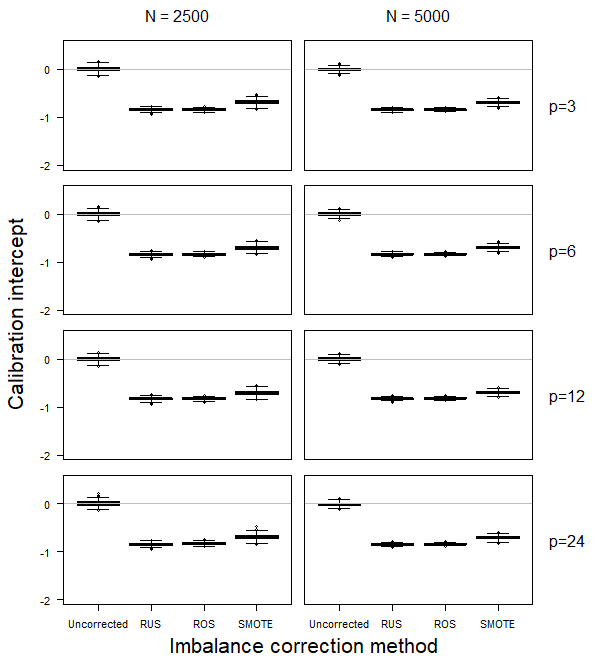


Figure S11. Test set calibration intercept for the Ridge models in the simulation scenarios with an event fraction of 1%, after recalibration of the models based on RUS, ROS, or SMOTE.


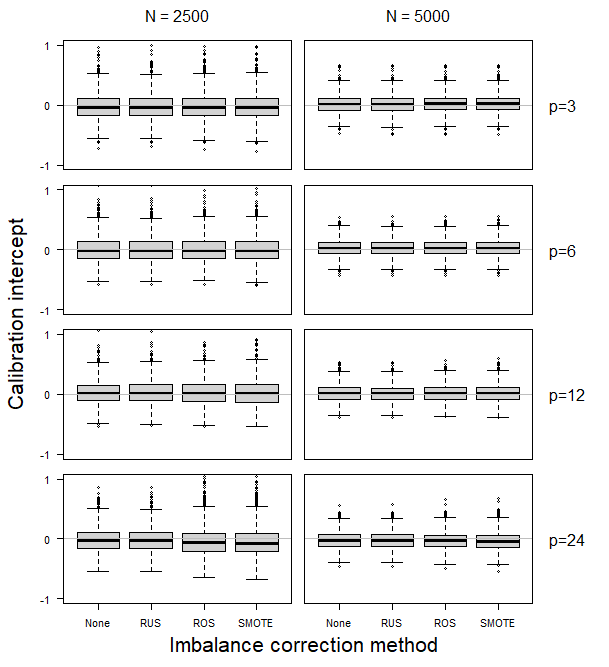


Figure S12. Test set calibration intercept for the Ridge models in the simulation scenarios with an event fraction of 10%, after recalibration of the models based on RUS, ROS, or SMOTE.


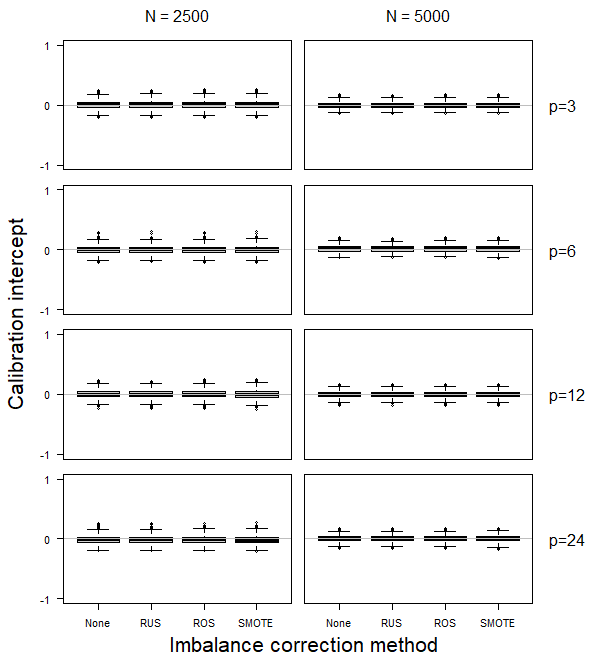


Figure S13. Test set calibration intercept for the Ridge models in the simulation scenarios with an event fraction of 30%, after recalibration of the models based on RUS, ROS, or SMOTE.


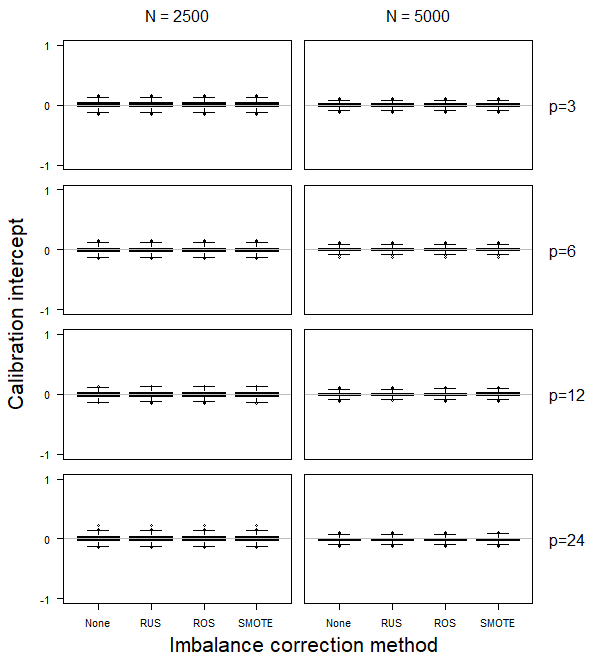


Figure S14. Test set calibration intercept for the SLR models in the simulation scenarios with an event fraction of 1%, after recalibration of the models based on RUS, ROS, or SMOTE.


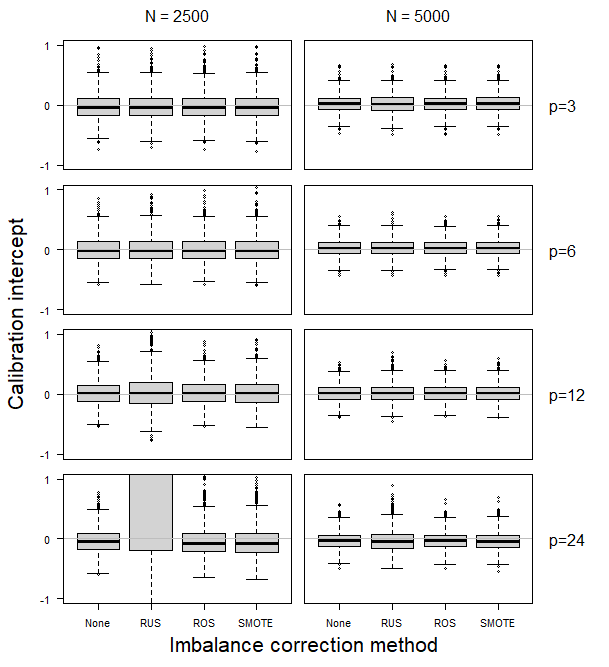


Figure S15. Test set calibration intercept for the SLR models in the simulation scenarios with an event fraction of 10%, after recalibration of the models based on RUS, ROS, or SMOTE.


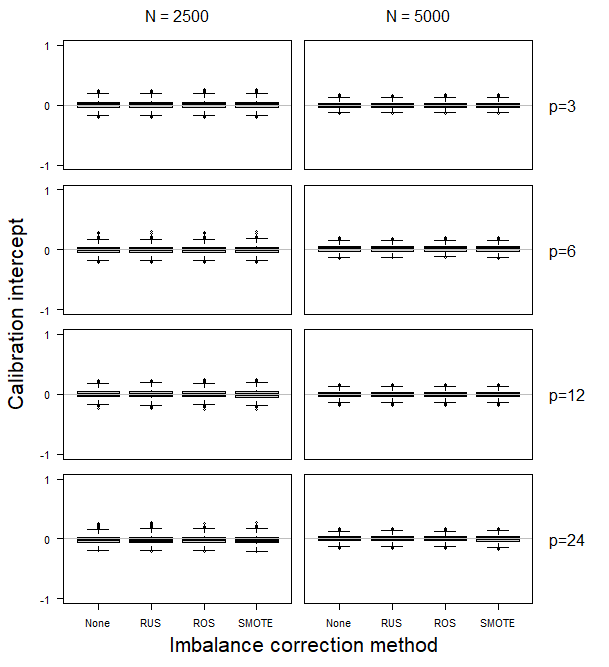


Figure S16. Test set calibration intercept for the SLR models in the simulation scenarios with an event fraction of 30%, after recalibration of the models based on RUS, ROS, or SMOTE.


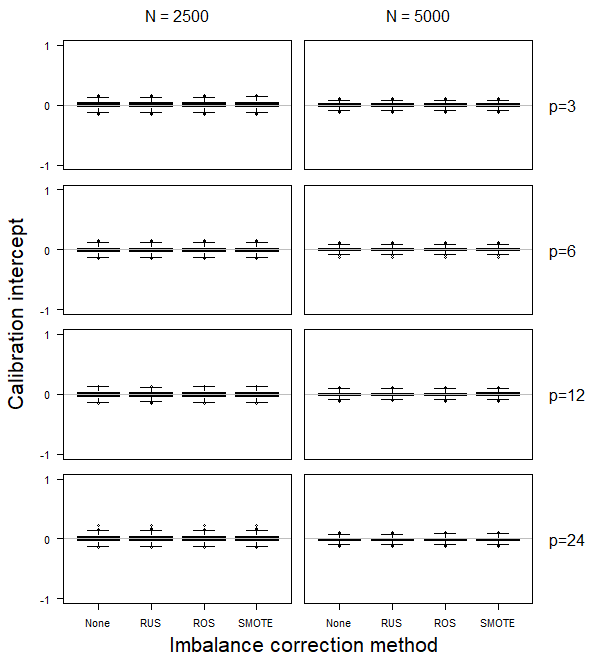


Figure S17. Test set calibration slope for the Ridge models in the simulation scenarios with an event fraction of 10%.


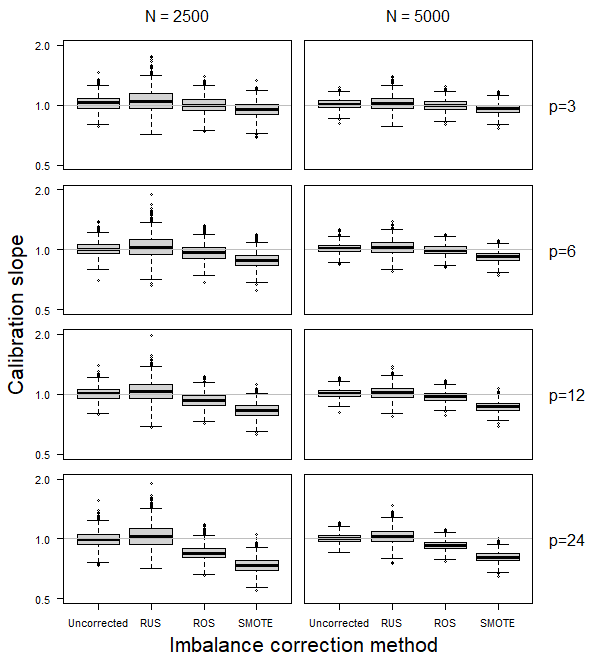


Figure S18. Test set calibration slope for the Ridge models in the simulation scenarios with an event fraction of 30%.


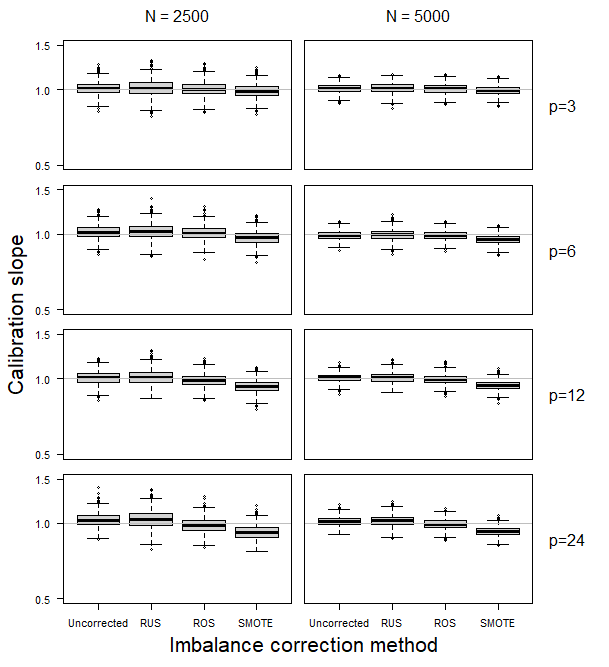


Figure S19. Test set calibration slope for the SLR models in the simulation scenarios with an event fraction of 1%.


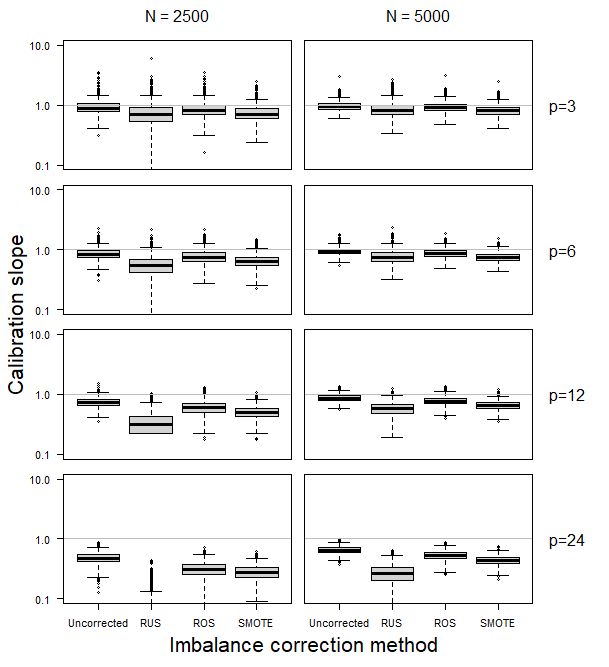


Figure S20. Test set calibration slope for the SLR models in the simulation scenarios with an event fraction of 10%.


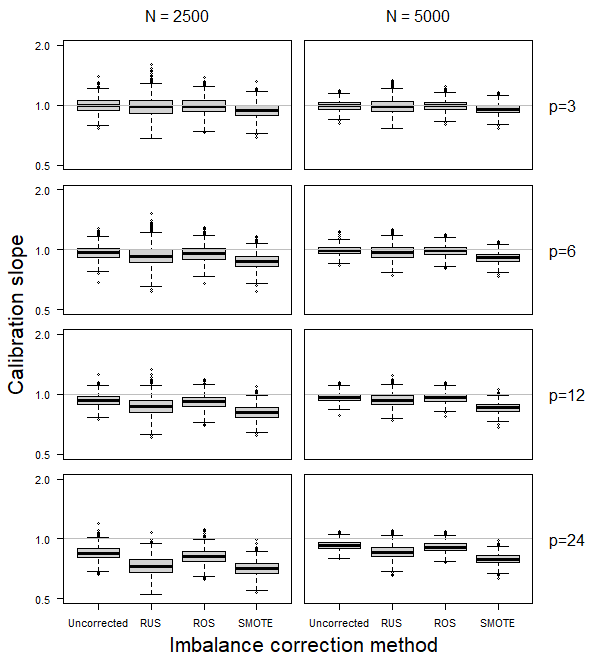


Figure S21. Test set calibration slope for the SLR models in the simulation scenarios with an event fraction of 30%.


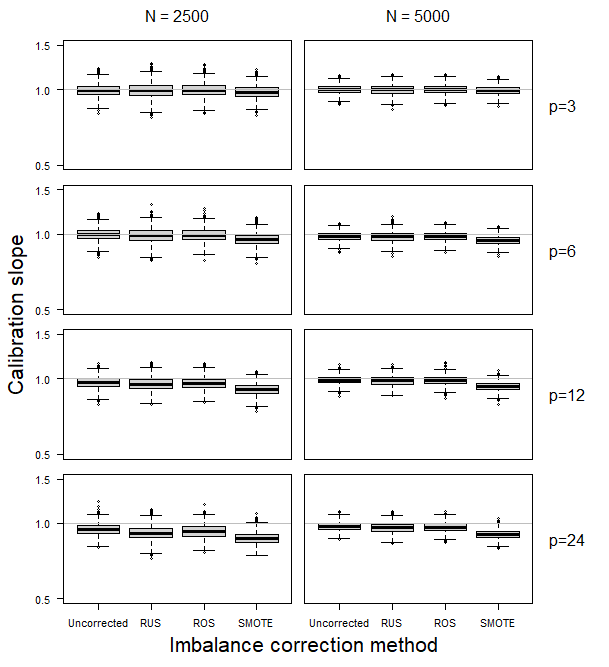


Figure S22. Test set classification accuracy for the Ridge models in the simulation scenarios with an event fraction of 1%. For uncorrected training sets, we used either the default threshold of 0.5 (“Unc .5”) or a threshold based on the true event fraction (“Unc EF”). For RUS/ROS/SMOTE, the default threshold of 0.5 was used.


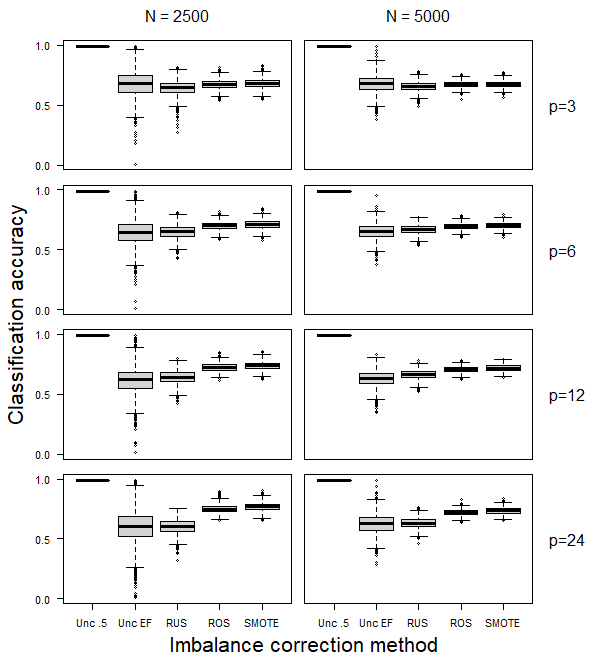


Figure S23. Test set classification accuracy for the Ridge models in the simulation scenarios with an event fraction of 10%. For uncorrected training sets, we used either the default threshold of 0.5 (“Unc .5”) or a threshold based on the true event fraction (“Unc EF”). For RUS/ROS/SMOTE, the default threshold of 0.5 was used.


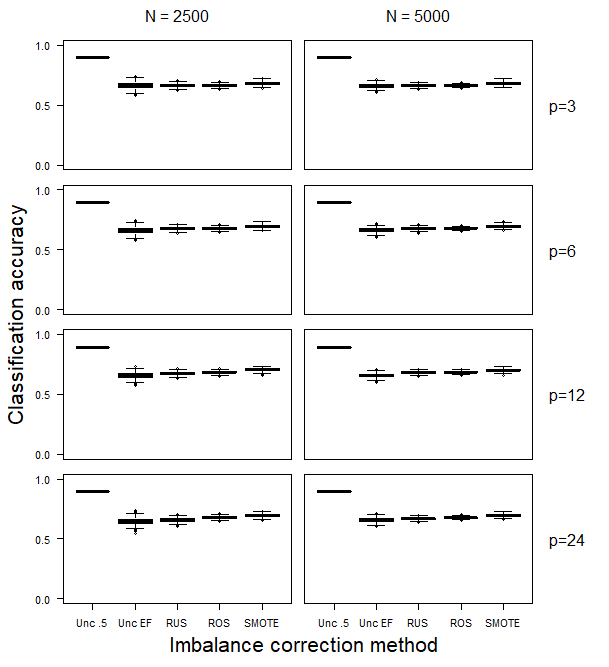


Figure S24. Test set classification accuracy for the Ridge models in the simulation scenarios with an event fraction of 30%. For uncorrected training sets, we used either the default threshold of 0.5 (“Unc .5”) or a threshold based on the true event fraction (“Unc EF”). For RUS/ROS/SMOTE, the default threshold of 0.5 was used.


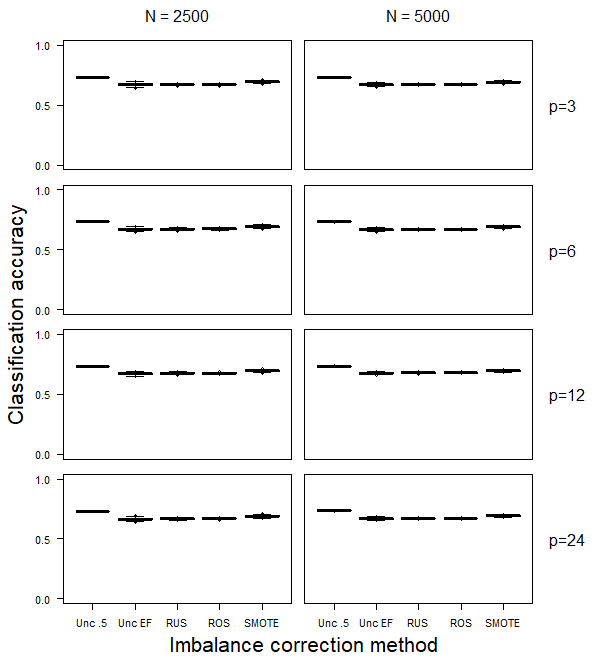


Figure S25. Test set classification accuracy for the SLR models in the simulation scenarios with an event fraction of 1%. For uncorrected training sets, we used either the default threshold of 0.5 (“Unc .5”) or a threshold based on the true event fraction (“Unc EF”). For RUS/ROS/SMOTE, the default threshold of 0.5 was used.


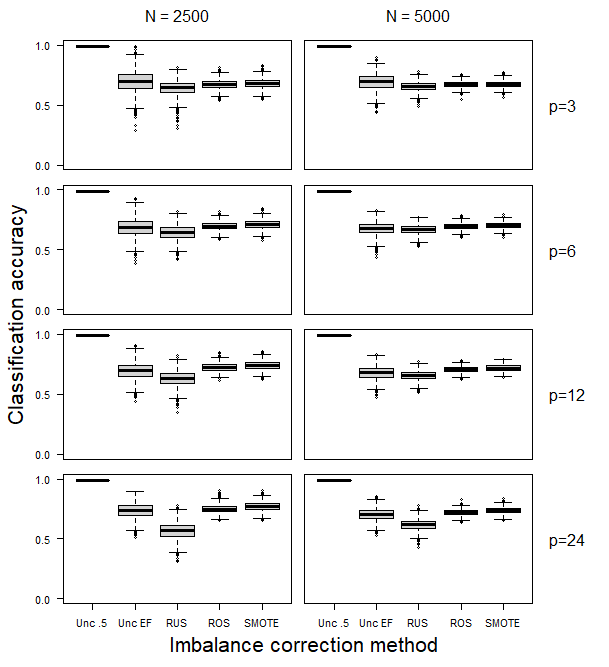


Figure S26. Test set classification accuracy for the SLR models in the simulation scenarios with an event fraction of 10%. For uncorrected training sets, we used either the default threshold of 0.5 (“Unc .5”) or a threshold based on the true event fraction (“Unc EF”). For RUS/ROS/SMOTE, the default threshold of 0.5 was used.


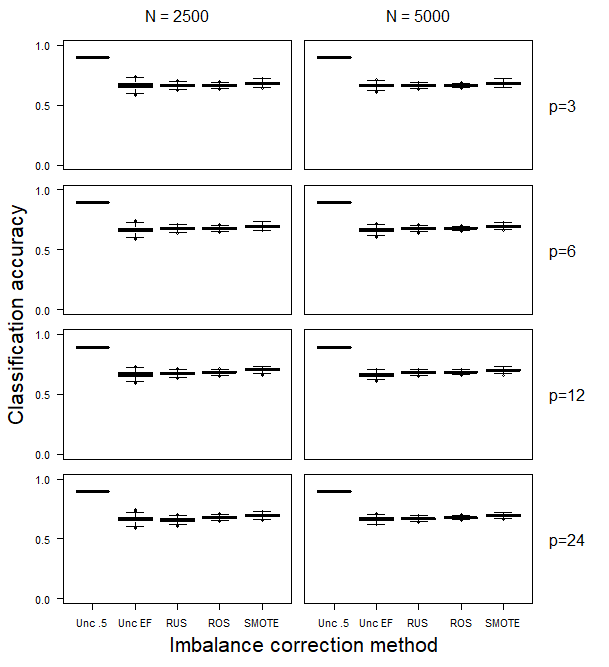


Figure S27. Test set classification accuracy for the SLR models in the simulation scenarios with an event fraction of 30%. For uncorrected training sets, we used either the default threshold of 0.5 (“Unc .5”) or a threshold based on the true event fraction (“Unc EF”). For RUS/ROS/SMOTE, the default threshold of 0.5 was used.


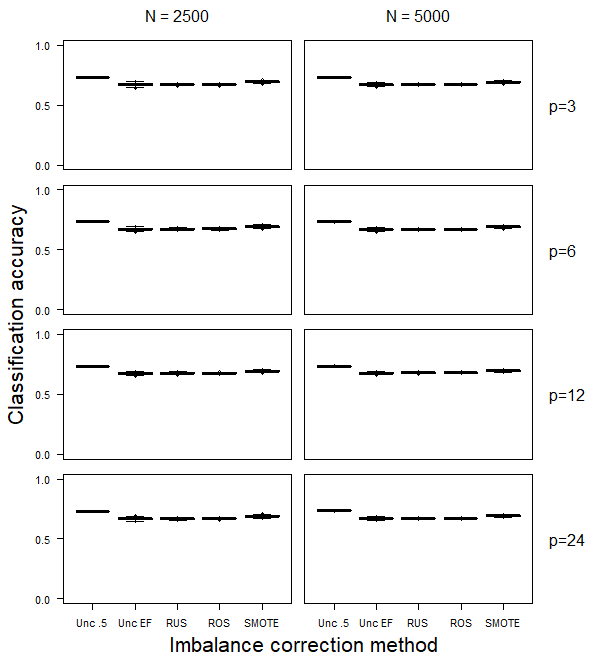


Figure S28. Test set sensitivity for the Ridge models in the simulation scenarios with an event fraction of 1%. For uncorrected training sets, we used either the default threshold of 0.5 (“Unc .5”) or a threshold based on the true event fraction (“Unc EF”). For RUS/ROS/SMOTE, the default threshold of 0.5 was used.


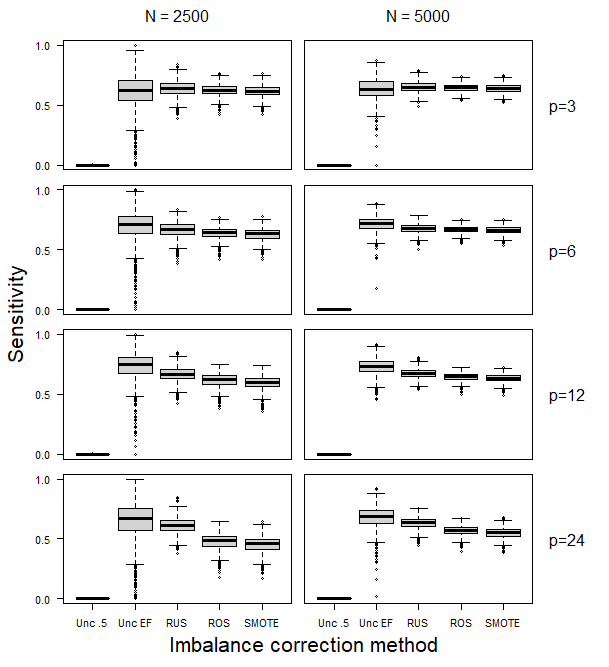


Figure S29. Test set sensitivity for the Ridge models in the simulation scenarios with an event fraction of 10%. For uncorrected training sets, we used either the default threshold of 0.5 (“Unc .5”) or a threshold based on the true event fraction (“Unc EF”). For RUS/ROS/SMOTE, the default threshold of 0.5 was used.


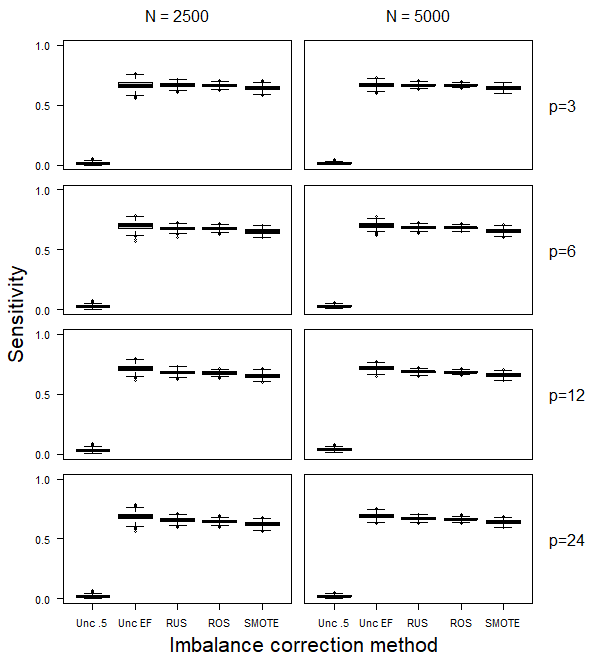


Figure S30. Test set sensitivity for the Ridge models in the simulation scenarios with an event fraction of 30%. For uncorrected training sets, we used either the default threshold of 0.5 (“Unc .5”) or a threshold based on the true event fraction (“Unc EF”). For RUS/ROS/SMOTE, the default threshold of 0.5 was used.


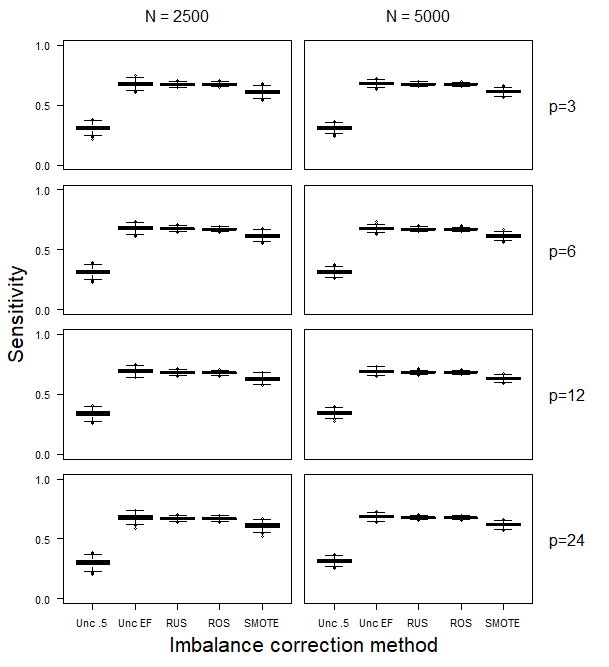


Figure S31. Test set sensitivity for the SLR models in the simulation scenarios with an event fraction of 1%. For uncorrected training sets, we used either the default threshold of 0.5 (“Unc .5”) or a threshold based on the true event fraction (“Unc EF”). For RUS/ROS/SMOTE, the default threshold of 0.5 was used.


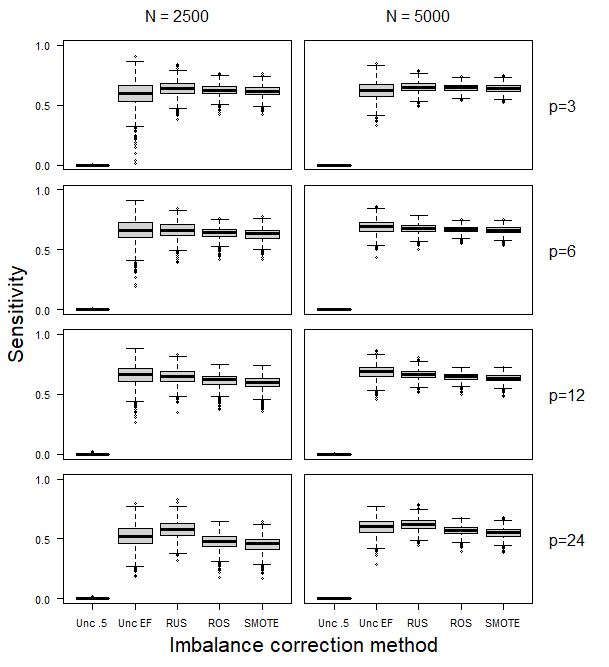


Figure S32. Test set sensitivity for the SLR models in the simulation scenarios with an event fraction of 10%. For uncorrected training sets, we used either the default threshold of 0.5 (“Unc .5”) or a threshold based on the true event fraction (“Unc EF”). For RUS/ROS/SMOTE, the default threshold of 0.5 was used.


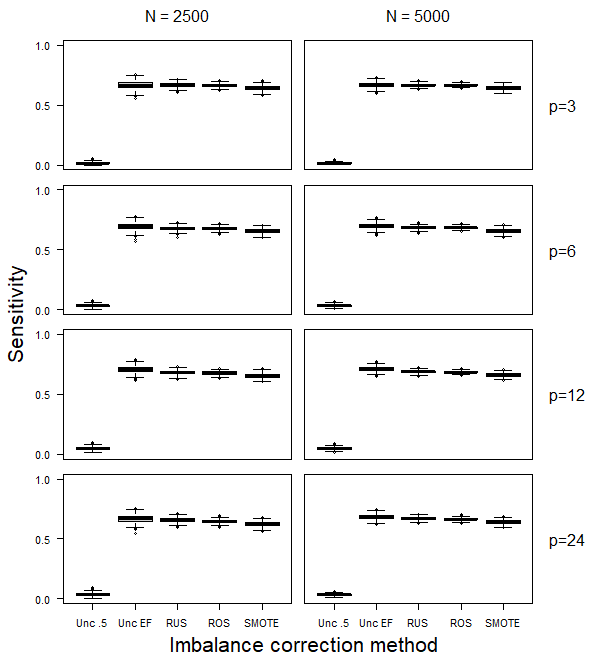


Figure S33. Test set sensitivity for the SLR models in the simulation scenarios with an event fraction of 30%. For uncorrected training sets, we used either the default threshold of 0.5 (“Unc .5”) or a threshold based on the true event fraction (“Unc EF”). For RUS/ROS/SMOTE, the default threshold of 0.5 was used.


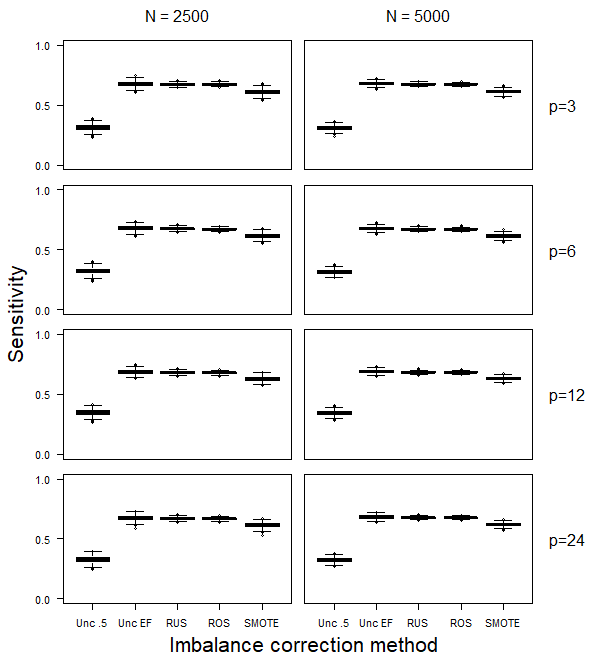


Figure S34. Test set specificity for the Ridge models in the simulation scenarios with an event fraction of 1%. For uncorrected training sets, we used either the default threshold of 0.5 (“Unc .5”) or a threshold based on the true event fraction (“Unc EF”). For RUS/ROS/SMOTE, the default threshold of 0.5 was used.


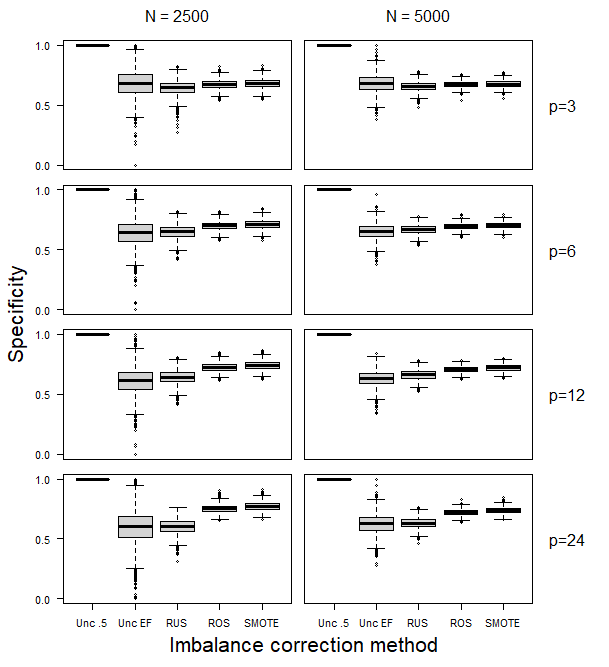


Figure S35. Test set specificity for the Ridge models in the simulation scenarios with an event fraction of 10%. For uncorrected training sets, we used either the default threshold of 0.5 (“Unc .5”) or a threshold based on the true event fraction (“Unc EF”). For RUS/ROS/SMOTE, the default threshold of 0.5 was used.


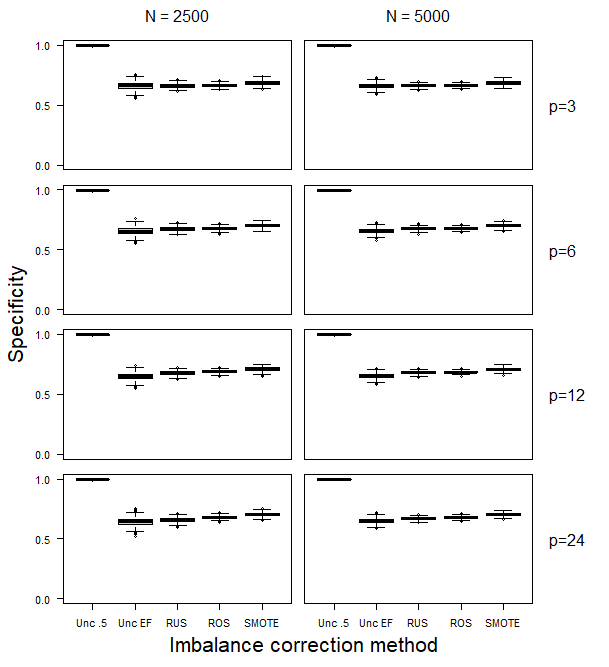


Figure S36. Test set specificity for the Ridge models in the simulation scenarios with an event fraction of 30%. For uncorrected training sets, we used either the default threshold of 0.5 (“Unc .5”) or a threshold based on the true event fraction (“Unc EF”). For RUS/ROS/SMOTE, the default threshold of 0.5 was used.


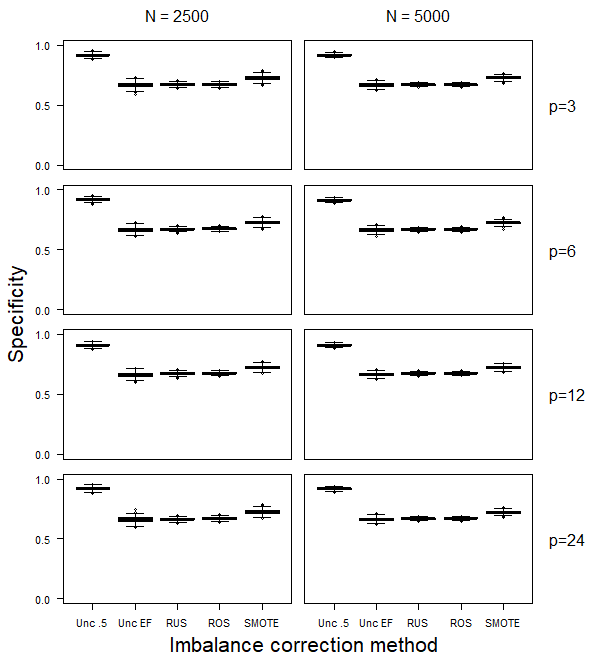


Figure S37. Test set specificity for the SLR models in the simulation scenarios with an event fraction of 1%. For uncorrected training sets, we used either the default threshold of 0.5 (“Unc .5”) or a threshold based on the true event fraction (“Unc EF”). For RUS/ROS/SMOTE, the default threshold of 0.5 was used.


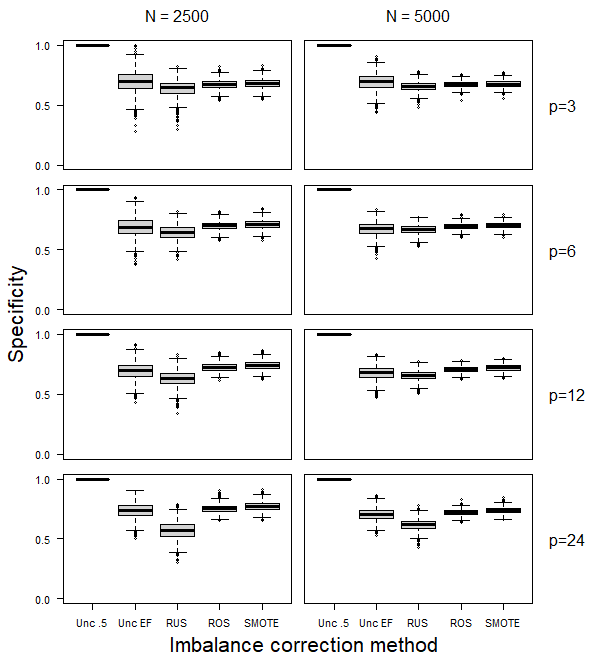


Figure S38. Test set specificity for the SLR models in the simulation scenarios with an event fraction of 10%. For uncorrected training sets, we used either the default threshold of 0.5 (“Unc .5”) or a threshold based on the true event fraction (“Unc EF”). For RUS/ROS/SMOTE, the default threshold of 0.5 was used.


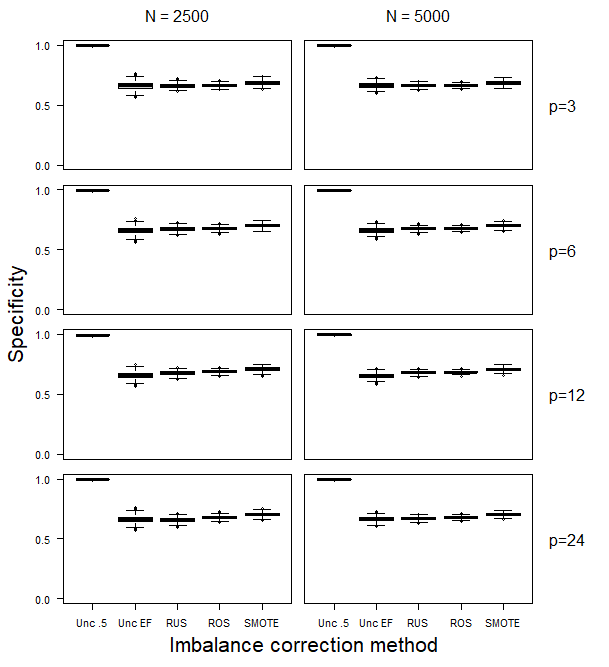


Figure S39. Test set specificity for the SLR models in the simulation scenarios with an event fraction of 30%. For uncorrected training sets, we used either the default threshold of 0.5 (“Unc .5”) or a threshold based on the true event fraction (“Unc EF”). For RUS/ROS/SMOTE, the default threshold of 0.5 was used.


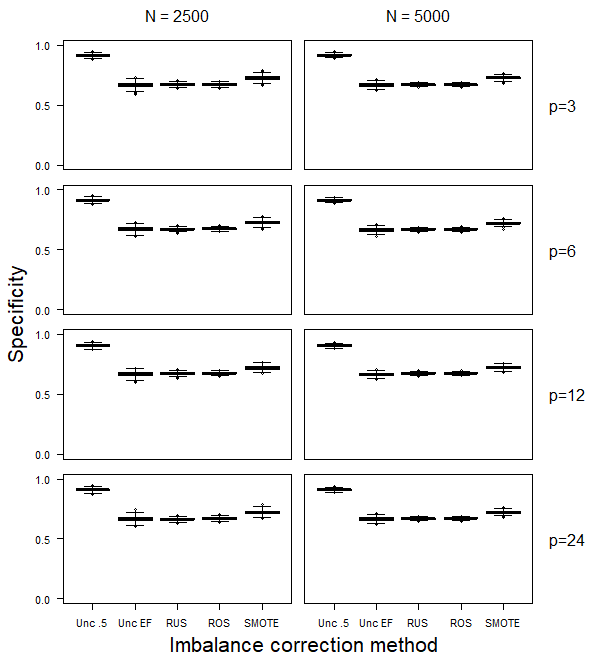

Supplement: ocac093_Supplementary_Data [file ocac093_supplementary_data.docx]
